# Density dependent regulation of inflammatory responses in macrophages

**DOI:** 10.3389/fimmu.2022.895488

**Published:** 2022-12-16

**Authors:** Alun Vaughan-Jackson, Szymon Stodolak, Kourosh H. Ebrahimi, Errin Johnson, Paul K. Reardon, Maeva Dupont, Shengpan Zhang, James S. O. McCullagh, William S. James

**Affiliations:** ^1^ James & Lillian Martin Centre, Sir William Dunn School of Pathology, University of Oxford, Oxford, United Kingdom; ^2^ Department of Chemistry, University of Oxford, Oxford, United Kingdom; ^3^ Sir William Dunn School of Pathology, University of Oxford, Oxford, United Kingdom; ^4^ Vagelos College of Physicians and Surgeons, Columbia University, New York, NY, United States

**Keywords:** macrophage, stem cell, inflammation, density, differentiation, microglia

## Abstract

Macrophage distribution density is tightly regulated within the body, yet the importance of macrophage crowding during *in vitro* culture is largely unstudied. Using a human induced pluripotent stem cell (iPSC)-derived macrophage model of tissue resident macrophages, we characterize how increasing macrophage culture density changes their morphology and phenotype before and after inflammatory stimulation. In particular, density drives changes in macrophage inflammatory cytokine and chemokine secretion in both resting and activated states. This density regulated inflammatory state is also evident in blood monocyte derived-macrophages, the human monocytic THP-1 immortalized cell line, and iPSC-derived microglia. Density-dependent changes appear to be driven by a transferable soluble factor, yet the precise mechanism remains unknown. Our findings highlight cell plating density as an important but frequently overlooked consideration of *in vitro* macrophage research relevant to a variety of fields ranging from basic macrophage cell biology to disease studies.

## 1 Introduction

Macrophages are a self-renewing essential component of the innate immune system. They play key roles in maintaining tissue homeostasis, by removing dead cells and remodeling the tissue environment, and in detecting and initiating an immune response to pathogens (1, 2). In general terms, there are two reservoirs of macrophages, blood monocyte derived macrophages and tissue resident macrophages. Tissue macrophages are long lived and can be found in every organ of the human body, within which they carry out specialized functions to maintain homeostatic functions of the tissue (3–5). These macrophages are ontogenically distinct, with monocyte-derived macrophages (MDM) continuously emerging from hematopoietic stem cells in the bone marrow, while many tissue macrophages are seeded before birth, originating from distinct embryonic precursors (excellently reviewed elsewhere (6)). However, many tissue macrophages are replenished during an individual’s lifespan by circulating monocytes. When specifically depleted from a tissue, alongside the infiltration of monocytes, tissue macrophages can also re-enter the cell cycle to repopulate the environment and regain their original distribution density within the tissue (7–10). Alternatively, during an active immune response, large numbers of monocytes infiltrate the tissue, attracted by cytokines and chemokines. As local density increases, so too can tissue damage and fibrosis (11). As such, density must also be able to decrease to resolve inflammation through suppression of the immune response and programmed cell death. Therefore, tissue macrophage distribution density is a tightly regulated system and feedback loops must exist to maintain optimal homeostasis (9, 10).

*In vitro* culturing of macrophages has been instrumental in elucidating feedback systems during inflammation. Most commonly used models utilize blood MDM or immortalized cell lines, but these cells lack features unique to tissue resident macrophages and often have varied metabolism and immune response profiles (10, 12, 13). Instead, we and others have shown that iPSC-derived macrophages (iPSC-Mac) share ontogeny with tissue macrophages and are, therefore, arguably a more suitable model for these cells (14, 15). For a long time *in vitro* macrophages were described as being in one of three states: M0 “resting”, M1 “inflammatory”, and M2 “anti-inflammatory/wound healing” (16, 17). However, macrophages are highly plastic cells that can respond differently to a broad array of pro- and anti-inflammatory stimuli. Recent multi-omics studies have demonstrated they can freely transition between these states and adopt identifiable features of both (18–20). Therefore, it is now more generally accepted that macrophages should be defined by the stimulus they are exposed to, along with factors such as the *in vivo* origin of the cells and how they are processed (21). The importance of individual factors will likely vary depending on the system, but it is certain that as advances in high throughput technologies allow us to define macrophages with greater precision, we will identify previously unconsidered modulators.

One such variable that has been largely overlooked is the number of cells cultured in a given area, the cell plating density, despite how much *in vivo* macrophage distribution density can fluctuate. Since the latter 20^th^ century, density has been known to influence non-macrophage cell growth by a feedback system called contact inhibition, which is mediated *via* receptor and cytoskeleton-dependent signaling through the Hippo pathway (22–25). Recently, density dependent feedback loops similar to bacterial quorum sensing have been proposed in macrophages. These are driven by accumulation of cytokines, reactive oxygen species, growth factors, and extracellular matrix components to influence their immune response (26–30). Alternatively, physical restriction of space has also been shown to reduce the macrophage inflammatory response (31). How important these systems are in relation to tissue resident *versus* monocyte or cell line-derived macrophages is unclear. It is crucial we understand more systematically the extent to which cell density regulates macrophage phenotype and function *in vitro*. In doing so, we will better understand how distribution density is maintained *in vivo*. Towards this goal, we here investigated whether density plays a role in regulating tissue macrophage homeostasis using human iPSC-Mac.

## 2 Materials and methods

### 2.1 iPSC lines

The derivation and characterization of the iPSC lines used in this study is described elsewhere: SFC840-03-03 (32), SFC841-03-01 (33) SFC856-03-04 (34)). All lines were derived from dermal fibroblasts from disease-free donors recruited through StemBANCC (Morrison et al., 2015) and the Oxford Parkinson’s Disease Centre: participants were recruited to this study having given signed informed consent, which included derivation of hiPSC lines from skin biopsies (Ethics Committee: National Health Service, Health Research Authority, NRES Committee South Central, Berkshire, UK, who specifically approved this part of the study (REC 10/H0505/71)). The iPSC lines were all derived using non-integrating Sendai reprogramming vectors (Cytotune, Life Technologies), cultured in mTeSR™1 (StemCell Technologies, #85850) on hESC-qualified Matrigel-coated plates (BD, #356234), passaging as clumps using 0.5 mM EDTA in PBS (Beers et al., 2012). Large-scale, low-passage frozen SNP-QCed batches were used for experiments to ensure consistency.

### 2.2 Cell culture: iPSC and iPSC-derived cells

All cells were incubated at 37°C, 5% CO_2_. Standard culture volumes for different plate formats were as follows: 10cm dish; 10 mL, 6-well; 2 mL, 12-well; 1 mL, 24 well; 0.5 mL, 96-well; 0.1 mL. iPSC were cultured in an in house alternative to commercial Essential 8 medium called OXE8 medium (35) on Geltrex™ (Gibco, #A1413201)-coated tissue culture dishes and passaged either as clumps using 0.5 mM EDTA in PBS, or using TrypLE™ Express (Gibco, #12604013). For 24 hours after TrypLE™ passaging, media was supplemented with 10 µM Y-27632 (Abcam, ab120129). Differentiation of iPSC into iPSC-Macs was carried out as previously described (35, 36) culturing in OXM medium and with the final 7 day differentiation occurring with cells plated at stated densities. iPSC-derived microglia were cultured based on a previously publication (37) by differentiation of iPSC-Mac precursor cells (PreMacs) for 14 days on fibronectin (Sigma, #F4759-1MG) coated tissue culture plates in RPMI (ATCC formulation, ThermoFisher, #A10491-01) supplemented with 100 ng/ml rhIL34 (Peprotech, #200-34-100uG), 25 ng/ml rhM-CSF (Life technologies, #PHC9501), 50 ng/ml rhTGF-β1 (Biolegend, #781804) and 1% Pen/Strep, with feeding every 3 days.

### 2.3 Cell culture: MDM isolation and differentiation

Human subject monocytes from healthy donors were provided by NHS Blood and Transplant services, under contract 17/WM/0333. Written informed consents were obtained from the donors before sample collection. Peripheral blood mononuclear cells (PBMC) were prepared by Ficoll gradient centrifugation followed by red blood cell lysis using ACK Lysis buffer (Gibco, #A1049201). After lysis, cells were divided for monocyte isolation either *via* adherence selection, or negative selection. To isolate by adherence, PBMCs were resuspended in RPMI 1640 (no serum) and ~1x10^7^ monocytes (assumption of 10% PBMCs are monocytes) plated in a 10 cm tissue culture dish and incubated for 2 hours at 37°C. Non-adherent cells were then removed by washing once with pre-warmed PBS. Remaining adherent monocytes were cultured for 2 weeks, with 100% media change every 3-4 days, in MDM media; RPMI medium (Sigma, #R8758) made up to 10% FBS (Sigma, #F9665), 1% GlutaMAX (100x, Gibco, #12634010), 1% HEPES (Gibco, #15630080), 1% Pen/Strep (Gibco, #15140122), and supplemented with 10 ng/mL recombinant human M-CSF. After 2 weeks, MDM were lifted by 15-minute incubation with TrypLE™ at 37°C and re-plated at desired densities and cultured for 2 more days. Alternatively, monocytes were isolated from PBMCs by magnetic bead negative selection using the human Pan Monocyte Isolation kit (Miltenyi Biotec, #130-096-537) according to manufacturer’s instructions. Monocytes isolated this way were then plated at desired densities and differentiated in MDM medium for 7 days with a 50% media change on day 4.

### 2.4 Cell culture: Immortalized cell lines

THP-1 monocytic cells (ATCC, #TIB-202) were cultured in RPMI (Sigma, #R8758) made up to 10% FBS, 1% GlutaMAX, 50 µM 2-mercaptoethanol (Gibco, #31350-010), and 1% Pen/Strep, with passaging every 3 days. For terminal differentiation into macrophages, THP-1s were treated with 5 ng/mL phorbol-12-myristate-13-acetate in culture medium for 48 hours. RAW264.7 (ATCC, #TIB-71) were maintained in DMEM (Sigma, #D6429) made to 10% FBS and 1% Pen/Strep and were passaged at 80-90% confluency (approximately every 3 days). Before plating for density assays, RAW cells were passaged twice into DMEM, 1% FBS, and 1% Pen/Strep to slow down growth.

### 2.5 Cell titration cytokine release assay

PreMacs were plated in a 96-well plate (Greiner, #655180) at the stated densities between 1x10^4^ and 1x10^5^ cells/well in 0.15 mL media and terminally differentiated for 7 days as described above, with the volume reduced to 0.1 mL on day 4. On day 7, 50% of media was removed and replaced with fresh medium containing 2x concentrated TLR agonist (LPS; InvivoGen, #tlrl-eklps. Pam3CSK4; InvivoGen, #tlrl-pms. TLR3; InvivoGen, #tlrl-pic) and cells were returned to 37C, 5% CO2 for 24 hours. Supernatant was then collected, spun at 400g for 5 minutes to remove dead/floating cells, and stored at -80°C until cytokine quantification. Assay was repeated as above with MDM, THP-1, and RAW264.7 cells with the following modifications. “Re-plate” MDM; stimulation 2 days after plating. THP-1; stimulation immediately after 2-day differentiation by PMA. RAW264.7; stimulation 2 days after plating.

### 2.6 Classical (M1) polarization

iPSC-Macs or MDM were cultured in 12-well plate format at stated densities. On the final day of differentiation, 100x concentration LPS and IFNγ in PBS were added directly to the well (10 µL, final concentration 100 ng/mL LPS and 20 ng/mL IFNγ (Gibco, # PHC4031)) and cells incubated overnight (16 hours). Supernatant was collected and spun 400g for 5 minutes to remove dead/floating cells and stored at -80°C until cytokine quantification. Cells were then washed with PBS and either lysed directly for RNA preparation (see below) or lifted by 10-15 minute incubation with Accutase™ Cell Dissociation Reagent (Stemcell technologies, #A1110501) (iPSC-Macs) or TrypLE™ (MDM) for immuno-staining and flow cytometry using antibodies and protocol as described previously (35).

### 2.7 Media exchange cytokine release assay

PreMacs were plated for terminal differentiation at stated densities as described for cell titration. On day 5 or day 6 of terminal differentiation (48 hours or 24 hours prior to stimulation), media was directly exchanged between densities, acting quickly to prevent prolonged exposure of iPSC-Macs to air which can encourage lifting. On day 7, cells were stimulated with 1 ng/mL LPS and supernatant collected for analysis as described for the cell titration assay.

### 2.8 Cytokine quantification: Enzyme-linked immunosorbent assay

Supernatants were thawed and diluted in water either 1:10 - 1:100 for quantification of TNFα (Invitrogen, #88-7346-88), 1:20 - 1:200 human IL-6 (Invitrogen, #88-7066-88), or 1:10 - 1:100 for mouse IL-6 (Invitrogen, #88-7064-88). Assay was run according to manufacturer’s instructions.

### 2.9 Cytokine quantification: Type I interferon

Supernatants were diluted 1:2 in advanced DMEM/F12 and type I interferon was assayed using a HEK-293 ISRE luciferase reporter cell line as previously described (38).

### 2.10 Cytokine quantification: Proteome profiler

Measurement of a panel of 36 cytokines, chemokines, and acute phase proteins was done using the Proteome Profiler Human Cytokine Array Kit (R&D Systems, #ARY005B). 1 mL of undiluted supernatant was used. The assay was run according to manufacturer’s instructions as instructed for use of Proteome Profiler Arrays with LI-COR Detection, as can be accessed at https://www.rndsystems.com/resources/technical/use-proteome-profiler-arrays-li-cor-detection. Arrays were visualized on the LI-COR Odyssey 9260 and quantified using Image Studio Lite version 5.2. A threshold of average signal (pixel density) normalized to the positive control ≥0.001 was chosen to distinguish unexpressed cytokines and chemokines which were not displayed if values below this threshold were observed across all 3 densities in both treatment groups.

### 2.11 Viability assay

Viability was determined in unstimulated, 1 ng/mL LPS, 1 µg/mL Pam3CSK4, or 3 µM Staurosporine (MP Biomedicals, #0219140080) stimulated cells. Cells were cultured as per cell titration assay at desired densities and stimulated by 50% media change with 2x agonist containing fresh media. After 24 hours, cells were stained with ReadyProbes™ Cell Viability Imaging Kit (Invitrogen, #R37609) diluted in Live Cell Imaging Solution (Invitrogen, #A14291DJ) for 15 minutes at room temperature. This was then replaced with fresh Live Cell Imaging Solution and cells were immediately visualised on the EVOS^®^ Fl Auto to quantify stained nuclei.

### 2.12 Microscopy

Low magnification, brightfield microscopy images were taken using the EVOS^®^ XL Core Imaging system. For confocal microscopy, PreMacs were plated for differentiation on 22mm glass slides. After 7 days, cells were fixed with 4% paraformaldehyde (Alfa Aesar, #J61899.AK) for 10 minutes followed by a PBS wash. Cells were then permeabilized for 1 hour at room temperature in PBS, 5% bovine serum albumin (Sigma, #A7906-100G), and 0.3% Triton X-100. They were then stained with Alexa Fluor™ 488 Phalloidin (Invitrogen, #A12379) diluted 1:400, and DAPI 1:1000, for 30 minutes in permeabilization buffer, followed by 3 washes in permeabilization buffer. Glass cover slips were then mounted onto slides with Dako fluorescence mounting medium (Agilent, #S3023), and imaged on the Olympus SoRa spinning disc microscope at 40x magnification. For scanning EM (SEM), cells were cultured on 15 mm glass cover slides. Cells were fixed first for 1 hour at room temperature in 2.5% glutaraldehyde in 0.1 M PIPES buffer at pH 7.4 followed by a wash in phosphate buffer and second fixation for 1 hour at 4°C with 1% OsO_4_ in 0.1 M PIPES Buffer. Samples were then washed 3 times with Milli-Q water for 5 minutes before dehydration. To dehydrate, samples were incubated for 5 minutes each in 50%, 70%, 90% and 95% ethanol (EtOH), and 3x10 minutes with 100% EtOH. Samples were then dried by incubating for 3 minutes in 1:1 EtOH: hexamethyldisilane (HMDS) followed by incubating twice for 2 minutes in pure HMDS. HMDS was then removed and fumes allowed to evaporate before mounting samples on carbon adhesive tape on an SEM stub and sputter coating with 15 nm gold. Samples were imaged using a Zeiss Sigma 300 SEM operated at 2 kV.

### 2.13 Adhesion assay

Macrophage adhesion across our 3 densities after transient exposure to Accutase™ was determined as previously reported (35).

### 2.14 RNA preparation and quantification: qPCR

Cells were lysed over ice in RLT buffer from RNeasy Mini Kit (QIAGEN, #74104) supplemented with 10 µL/mL β-mercaptoethanol. Lysates were homogenized using the QIAshredder kit (QIAGEN, #79656) and stored at -80°C until extraction. All RNA extractions were done using the RNeasy Mini Kit (QIAGEN, #74104) including the optional DNase treatment step (RNAse-Free DNase Set, QIAGEN, #79254) and eluted in 35-50 µL RNAse-free water. RNA concentration was quantified with the Nanodrop 2000c. cDNA was made using the High-Capacity RNA-to-cDNA™ Kit (Applied Biosystems, #10704217). For each experiment, equal quantity of RNA was added across samples, using up to 9µL RNA. qPCR reactions comprised of 1 volume sample cDNA to 3 volumes master mix (Power SYBR™Green PCR Master Mix (Applied Biosystems, #4367659), forward and reverse primer mix, and nuclease-free water at a ratio of 5 µL: 1 µL: 2 µL respectively). Primers used are listed in **supplementary table 5**. Reactions were in run in triplicate in 384-well format, 6-8 µL/sample in the Applied Bioscience QuantStudio™ 5. Default cycling conditions were used (**supplementary table 6**). Analysis of gene expression was performed by normalization of Ct values to the average Ct value of two housekeeper genes, *VIPAR* and *UBE4A*, and displayed as fold difference as calculated by 2^-ΔCt^.

### 2.15 RNA preparation and quantification: RNA-Seq

RNA-Seq data was generated by Novogene. RNA was extracted in triplicate from a total of 1.7x10^6^ iPSC-Macs (SFC840-03-03 cell line) across each density cultured in 6-well plate format, using the RNeasy Mini Kit, including optional DNase treatment step. Sample handling, library preparation, and sequencing were carried out as previously reported (35).

### 2.16 RNA-Seq analysis

Pre-processing, mapping, and quantification was carried out as previously reported (35). All downstream analyses were done using the R 4.0 programming language (R Core Team, 2020). Transcript abundance estimates in TPMs were summarized to the gene level using the tximport 1.16.0 package to correct for sample specific transcript length biases (39). Lowly expressed genes with length-scaled abundance estimates less than 10 in 3 samples were excluded by the default filtering function in the edgeR package (40). Differential expression was tested using limma-voom (41). Time at harvesting macrophages from a factory was defined as a batch variable in the design. GO enrichment analysis was conducted using the EGSEA package by leveraging 12 prominent gene set testing algorithms to obtain a consensus rank for each GO term (42). GO terms with less than 10 genes expressed in our dataset were excluded for a more stringent enrichment analysis. The background gene set used for the analysis was the total number of the unique genes observed in the experiment. KEGG analysis was done on differentially expressed genes, with all expressed genes as background, using the kegga function from the limma package. A publicly available dataset of human MDM, iPSC-Mac, iPSC-microglia, and M1 or M2 polarized iPSC-Mac and MDM was pre-processed as above and used as a reference (43). The gene expression values from both studies were corrected for library size using the fpm function from the DESeq2 package. Batch effects were estimated using surrogate variable analysis and subsequently removed with the limma removeBatchEffect function (44). The samples from this study were then projected onto a principal component analysis (PCA) space generated by the samples from the reference study to visualize the level of transcriptomic similarity. 3D volcano and radial plots were produced with the volcano3D package (45).

### 2.17 Phagocytosis assay

Macrophages were cultured at desired densities in a 24-well plate. 2.5, 5, and 10 μg Zymosan A (S. cerevisiae) BioParticles™ Alexa Fluor-488 (ThermoFisher, #Z23373) were added to low, mid, or high density cells respectively by 50% media change and incubated for 1 hour or 4 hours 37°C, 5% CO2. Particles not taken up were quenched with 0.025% (v/v) Trypan Blue (Sigma, #T8154) in PBS before lifting the cells by 15-minute incubation with Accutase™. Cells were fixed with 4% paraformaldehyde and fluorescence measured by flow cytometry with the BD LSRFortessa™ X-20.

### 2.18 Analysis of polar and ionic metabolites

Anion-exchange chromatography-mass spectrometry (IC-MS) was used for analysis of polar and ionic metabolites in the media samples as described in detail previously (46). We used compound discover software (Thermofisher Scientific) for data processing and statistical analysis. Metabolites were identified using predicted composition and searches against four global database MzCloud, Metabolika, ChemSpider, and an in-house database of authentic standards that included retention times. Matches to the in-house database were: retention time: true, RT (retention time) tolerance 2, and mass tolerance 5 ppm. Peak areas for the extracted ion chromatogram (EIC) for each identified metabolite are presented as a relative measure of abundance. To estimate the levels of itaconate and taurine, a standard curve was prepared. Standard samples were prepared in the medium used for growing the cells and all samples were measured using IC-MS. Analysis of data and calculations of EIC peak areas were performed using MestReNova software.

### 2.19 Analysis of amino acids

The AccQ∙Tag ultra derivatization kit (Waters) was used according to the manufacturers protocol to chemically derivatize amino acids present in the media samples. Samples were analyzed using Thermo Utimate 3000 UHPLC system coupled directly to a Q-Exactive Hybrid Quadrupole-Orbitrap mass spectrometer as described in detail previously (47). Data collection was performed in positive ion mode. Progenesis QI (Non-Linear Dynamics, Elstree, UK) software was used to analyze data with thresholds settings of p < 0.05 and FC > 1.2. Area under the curve for extracted ion chromatograms was used as a measure of relative metabolite abundance between samples.

### 2.20 IL-10 neutralization assay

Macrophages were cultured as described above for 6 days. On day 6, 25% of the media was replaced with fresh media containing IL-10 neutralizing antibody (R&D, #MAB217-100), an isotype control (R&D, # MAB002), or recombinant human IL-10 (Peprotech, #200-10) so that the final stated concentrations were achieved, Macrophage were then cultured for a further 24 hours before then being stimulated with Pam3CSK4 and TNFα secretion into the supernatant analysed as described above.

### 2.21 Statistical analysis

Specific statistical analysis of data is described in the figure legends. In general, normality of data was tested by Shapiro-Wilk test. Normally distributed data group comparisons were made using either two-tailed t-test, one-way or two-way ANOVA, or mixed effects analysis when repeated measure values were missing, as appropriate. Where multiple variables were measured from the same sample, Greenhouse-Geisser correction was applied. When not normally distributed, a two-sided Wilcoxon test was applied. Results with P < 0.05 were considered significant, and significance was defined as * <0.05, ** <0.01, ***<0.001, ****<0.0001. Outside of transcriptomic and metabolomic analysis, data analyses were performed using GraphPad Prism v9 and Microsoft Excel.

## 3 Results

### 3.1 iPSC-Mac cytokine and chemokine secretion is dependent on plating density

**Figure 1:**
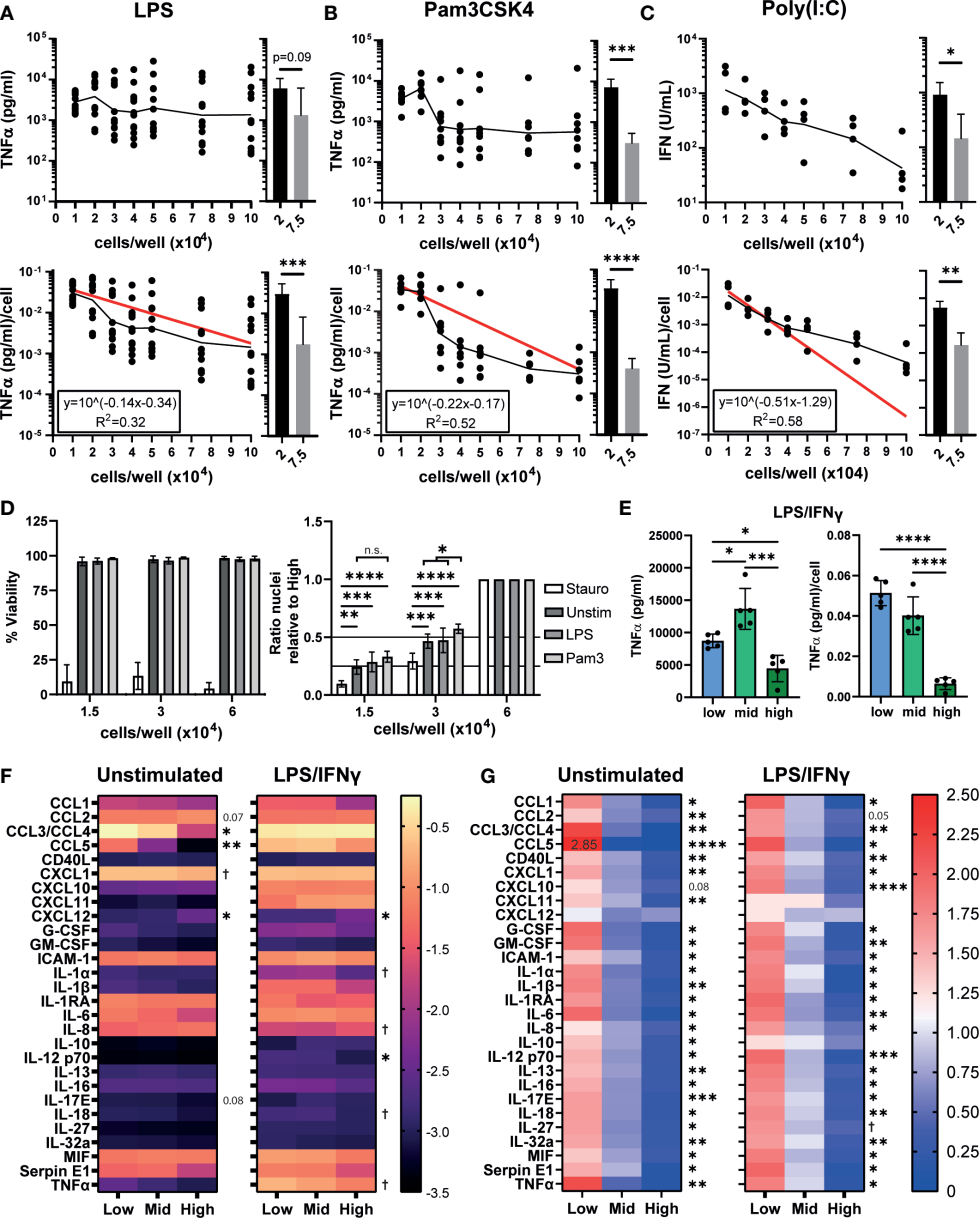
iPSC-Mac cytokine and chemokine secretion is dependent on plating density. **(A, B)** Secretion of TNFα determined by ELISA or **(C)** type I IFN determined by THP-1-reporter assay, 24 hours post stimulation from increasing densities of iPSC-Macs stimulated with **(A)** 1 ng/mL LPS (TLR4 agonist) **(B)** 1 µg/mL Pam3CSK4 (TLR2) or **(C)** 1 µg/mL Poly(I:C) (TLR3). Top: total concentration in 100 µL of supernatant. Bottom: concentration normalised to number of cells plated. **(A-C)** Scatter plot displays geometric mean (black line) and non-linear regression curve fit (red) for normalised values. Histograms display mean +/- standard deviation (SD). Significance calculated by pairwise comparison of 2x10^4^ *vs* 7.5x10^4^ cells/well; **(A)** n=12 across 3 independent iPSC lines, Wilcoxon matched-pairs signed rank test, **(B)** n=7, 3 iPSC lines, ratio paired T-test **(C)** n=4, 1 iPSC line, ratio paired T-test. **(D)** Density effect is not due to changes in cell number after plating or stimulation. Left; percentage viable cells, right; relative total number of nuclei normalized by division against the high density condition. Cell stimulated with 3 µg/mL Staurosporine, 1 ng/mL LPS or 1 µg/mL Pam3CSK4. Mean +/- SD. n=4, 2 iPSC lines, mixed effects analysis, Tukey’s multiple comparisons test. **(E)** TNFα secretion after polarization by 100 ng/mL LPS and 20 ng/mL IFNγ for 24 hours. Left; total, right; normalized to plated cell number. Mean +/- SD of n=5, 1 iPSC line, repeated measures one-way ANOVA, Tukey’s multiple comparison test. **(F, G)** Most macrophage cytokines and chemokines are influenced by density. **(F)** Total cytokine and chemokine secretion or **(G)** secretion normalized against the mean of all 3 densities, in unstimulated iPSC-Macs or polarized with LPS and IFNγ. **(F)** scale units represent the arbitrary unit pixel density normalized to a positive control (see methods), Y=Log_10_ (y) transformed. **(G)** scale is ratio relative to the group mean. **(F, G)** n=3, 1 iPSC line, two-way ANOVA, Tukey’s multiple comparisons test with Greenhouse-Geisser correction. Significance shown for the low *vs* high comparison, except where † indicates low *vs* high was not significant but mid *vs* high comparison 0.01< p <0.05. In all other cases, significance is defined as * <0.05, ** <0.01, ***<0.001, ****<0.0001.

Plating densities stated in publications can vary from as low as ~9x10^3^ cells/cm^2^ to as high as 4x10^5^ cells/cm^2^, if they are even reported (**Figures S1A, B**, **Supplementary Table 1**), and changes in density have been reported to alter human macrophage responses to Toll-like receptor (TLR) stimulation (29, 31, 48). We first determined whether similar changes occur to cytokine secretion by iPSC-Macs. iPSC-Macs were plated at increasing densities and stimulated with lipopolysaccharide (LPS, TLR4 agonist), Pam3CSK4 (TLR1/2), or Polyinosinic:polycytidylic acid (Poly(I:C), TLR3) for 24 hours. The amount of tumor necrosis factor-alpha (TNFα) secreted per LPS stimulated cell was inversely related to the cell density (**Figure 1A** bottom), resulting in a highly variable but fairly level overall concentration in the culture supernatant (**Figure 1A**, top). Likely causes of this variability include small variations in true cell number at the time of stimulation, as well as intrinsic variables in the iPSC-Mac differentiation protocol such as culture age and iPSC donor. This density effect was clearer after Pam3CSK4 stimulation, where total TNFα and TNFα/cell clearly decreases as density increases >2x10^4^ cells/well (**Figure 1B**). This decline was not unique to TNFα, as a change in IL-6 secretion per cell was also observed (**Figures S1C, D**). Type I interferon secretion post TLR3 stimulation was also significantly reduced (**Figure 1C**). Inflammatory stimulation can induce cell death *via* inflammasome activation resulting in apoptosis or necroptosis (49). To test whether decreased cytokine production by high density cells is due to increased cell death, viability was compared between unstimulated, TLR stimulated, or staurosporine (an apoptosis inducer) treated iPSC-Mac. For this we selected three densities corresponding to 1.5, 3, and 6x10^4^ cells/well in a 96 well plate (0.44, 0.88, and 1.76x10^5^ cells/cm^2^ respectively). We will hereon refer to these as “low”, “mid” and “high” density cells (**Figure S1A**). While total percentage of viable iPSC-Macs was reduced by staurosporine, TLR stimulation did not cause noticeable changes (**Figure 1D**, left) and no change in relative total cell number between densities was observed as determined by the number of nuclei present (**Figure 1D**, right). Total number of nuclei following Pam3CSK4 stimulation was significantly increased at mid density *versus* untreated control, but not at low densities, likely reflecting variability within the assay (*e.g.* cells lost during wash steps) rather than functional differences.

Typically, inflammatory macrophages are described as classically activated or “M1 macrophages”, which can be generated by *in vitro* polarization using a combination of LPS and IFNγ (50). We assessed whether TNFα secretion after M1 polarization is also density dependent. While low and mid density “M1” cells secreted similar concentrations of TNFα/cell post-stimulation, high density cells secreted significantly less (**Figure 1E**). We next sought to determine whether other cytokines or chemokines are influenced by cell density. We have previously reported that our iPSC-Macs produce several cytokines constitutively under resting conditions (35). Therefore, we also checked whether this resting state is influenced or explained by plating density. Indeed, unstimulated iPSC-Macs of all densities constitutively secreted CCL2, CCL3/4, CXCL1, ICAM-1, IL-1RA, IL-6, IL-8, MIF, and Serpin E1 (**Figure 1F**, left). However, low density cells secrete significantly more CCL3/4 (low: -0.271 ± 0.071 arbitrary units (AU), Log_10_ transformed data, high: -1.714 ± 0.242 AU) and CCL5 (low: -1.254 ± 0.098 AU, high: -3.364 ± 0.098 AU) than high density cells. High density cells only secreted more CCL2 (low: -1.165 ± 0.130 AU, high: -1.019 ± 0.141 AU) and CXCL12 (low: -2.935 ± 0.187 AU, high: -2.509 ± 0.249 AU). However, per cell, low density cells still produce more CCL2 than high (low: 1.622 ± 0.038 AU, values normalized against the group mean, high: 0.561 ± 0.053 AU), and only CXCL12 is not significantly differentially secreted between densities (low: 1.345 ± 0.191 AU, high: 0.883 ± 0.130 AU, p = 0.206) (**Figure 1G**, right). After stimulation with LPS/IFNγ, a majority of cytokines and chemokines measured were upregulated across densities (**Figures 1F**, left, **S1E**). Most notable of these were CCL5, CXCL10, CXCL11, IL-1β, and TNFα (**Figure S1E**). IL-1RA and IL-8 meanwhile were down regulated. With CCL3/4, low density cells displayed no change in secretion, whereas secretion increased approximately 5-fold from high density cells (**Figure S1E**). Again, low density cells still secreted more cytokines and chemokines per cell than high (**Figure 1G**, right) with the exception of CXCL12 (low: 1.017 ± 0.311 AU, high: 0.933 ± 0.097 AU), CXCL11 (low: 0.887 ± 0.378 AU, high: 0.843 ± 0.418 AU), and IL-10 (low: 1.032 ± 0.247 AU, high: 0.760 ± 0.204 AU). Overall, we find that iPSC-Macs plated at low densities have an increased inflammatory cytokine and chemokine profile under resting and M1-polarized conditions.

### 3.2 iPSC-Macs plated at different densities have different morphologies

**Figure 2:**
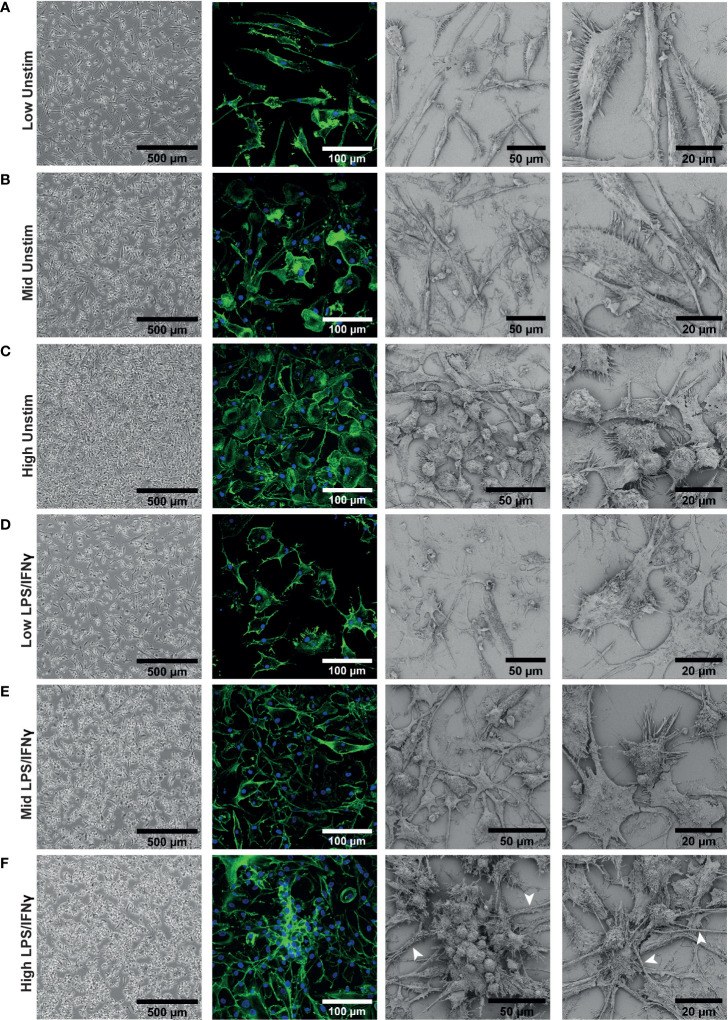
iPSC-Macs plated at different densities have different morphologies. Representative images of iPSC-Mac: Far left; brightfield with no fixation or staining. Middle left; confocal, 40x magnification, stained for nuclei (DAPI, blue) and actin (Phalloidin, green). Right; Scanning EM. **(A–C)** Unstimulated cells. **(D–F)** iPSC-Macs 24 hours after M1 polarization. **(F)** Arrows indicate potential tunnelling nanotube-like structures.

We next investigated whether there were other density dependent phenotypic differences between our iPSC-Macs. Differentiated macrophages showed obvious morphological differences by brightfield microscopy. Low density cells had an elongated bipolar morphology (**Figure 2A**). As density increases, these protrusions shrank, and more rounded cells were apparent (**Figures 2B, C**). This was more clearly observed with confocal and electron microscopy. Confocal microscopy revealed that differences in cell structure corresponded to differences in actin cytoskeleton. Electron microscopy showed that at high density, morphology was heterogeneous, with both flattened and rounded, non-polar cells visible (**Figure 2C**). Despite these structural differences, cells were equally adherent across densities as determined by sensitivity to enzymatic lifting (**Figure S2**). This would suggest that the rounded cells seen at high density are not undifferentiated, non-adherent cells.

After LPS/IFNγ polarization, low density cells did not always show obvious changes in morphology with brightfield microscopy (**Figure 2D**), but confocal and electron microscopy revealed that they appear to become more flattened and multipolar. Mid and high-density cells displayed clearer changes, projecting multiple processes and forming clusters of cells (**Figures 2E, F**). These clustered cells were not fused, as has been reported with M2-like cells (51, 52). In addition, we observed by confocal and electron microscopy thick tube-like structures reminiscent of tunnelling nanotubes in the high density environment (53) (**Figure 2F**). In summary, low density cells appeared to have a larger surface area and were typically more bipolar. High density cells responded more noticeably to inflammatory stimulation, with considerable direct cell-cell interactions occurring.

### 3.3 Density influences surface marker phenotype

**Figure 3:**
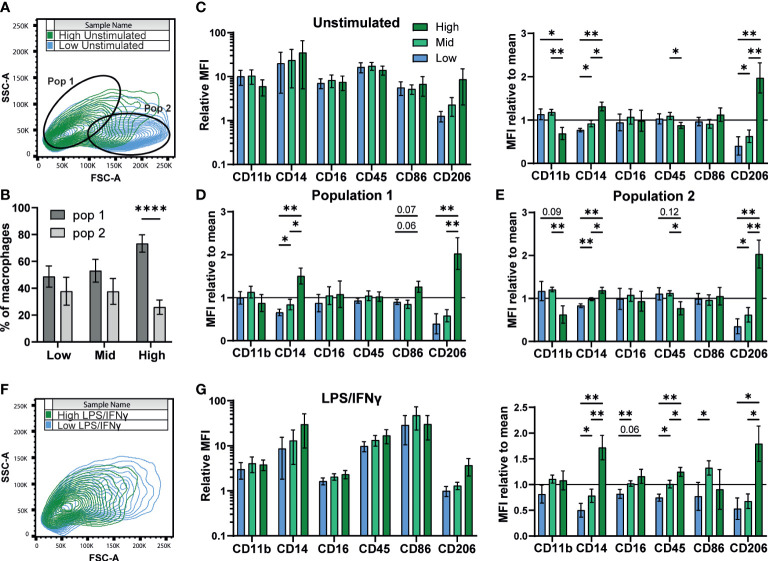
Density influences surface marker phenotype dependent and independent on morphology. **(A, B)** iPSC-Macs separate out into two populations measurable by comparing shape and granularity by flow cytometry. **(B)** n=5, 1 iPSC line, Two-way ANOVA, Sidak’s multiple comparisons test. **(C-E)** Expression of macrophage markers by different density resting iPSC-Macs. **(C)** Left; Mean fluorescence intensity (MFI) calculated by normalization of fluorescence against an isotype control. Right; MFI normalized by dividing by the mean of all 3 densities. **(D)** MFI normalized to the group mean subgating for population 1 and **(E)** population 2. **(F)** Differences in size and granularity are lost after polarization. **(G)** Left; MFI, and right; MFI normalized to the group mean, of macrophage markers on polarized iPSC-Macs. **(C-G)** n=5 (CD86 n=4), 1 iPSC line, mixed effects analysis, Tukey’s multiple comparisons test with Greenhouse-Geisser correction. Significance is defined as * <0.05, ** <0.01, ****<0.0001.

A previous study of murine bone marrow derived macrophage differentiation reported that density influences surface expression of markers such as CD11b (54). To test if density influences the differentiation state of iPSC-Macs, we measured expression of several typical macrophage surface markers; CD11b, CD14, CD16, and CD45. We also compared two markers associated with M1 and M2 polarization, the co-stimulatory molecule CD86 and the mannose receptor CD206, respectively (50, 55). Flow cytometry analysis identified two observable populations by scattering (Pop 1 and Pop 2, **Figure 3A**). While low and mid density cells were evenly distributed between these populations, high density cells were smaller, belonging predominantly to Pop 1 (FSC-A^low^ SSC-A^high^, **Figure 3B**) (56). Surface marker immuno-staining showed clear expression of all markers above isotype control levels (**Figure 3C** left, **S3A**). When normalized against the pooled mean of all three densities, expression of CD14 and CD206 were both significantly higher on high density cells, while CD11b and CD45 showed trends towards higher expression in low and mid density cells (**Figure 3C**, right). However, when sub-gated by population, this difference in CD11b and CD45 was only significant in Pop 2, suggesting a relation to cell size (**Figures 3D, E**). CD14 and CD206 on the other hand were significantly higher regardless of population and are therefore independent of cell morphology. Once stimulated with LPS/IFNγ, scattering of iPSC-Mac of all densities became more uniform (**Figure 3F**). CD11b, CD16, and CD206 expression all declined while CD14 and CD45 expression only dropped in low and mid density cells, but not high density cells (**Figure 3G**, left, **S3B, C**). Overall, high density cells expressed significantly higher CD14, CD16, CD45, and CD206 (**Figure 3G**, right). CD86 surface expression increased for all iPSC-Mac, indicating successful M1 polarization, but no clear trend between densities was observed (**Figure 3G**, right, **S3**). These results indicate that iPSC-Mac surface antigen composition and cell morphology are independently influenced by plating density.

### 3.4 Low density drives a pro-inflammatory transcriptional signature while high density favors phagocytosis

**Figure 4:**
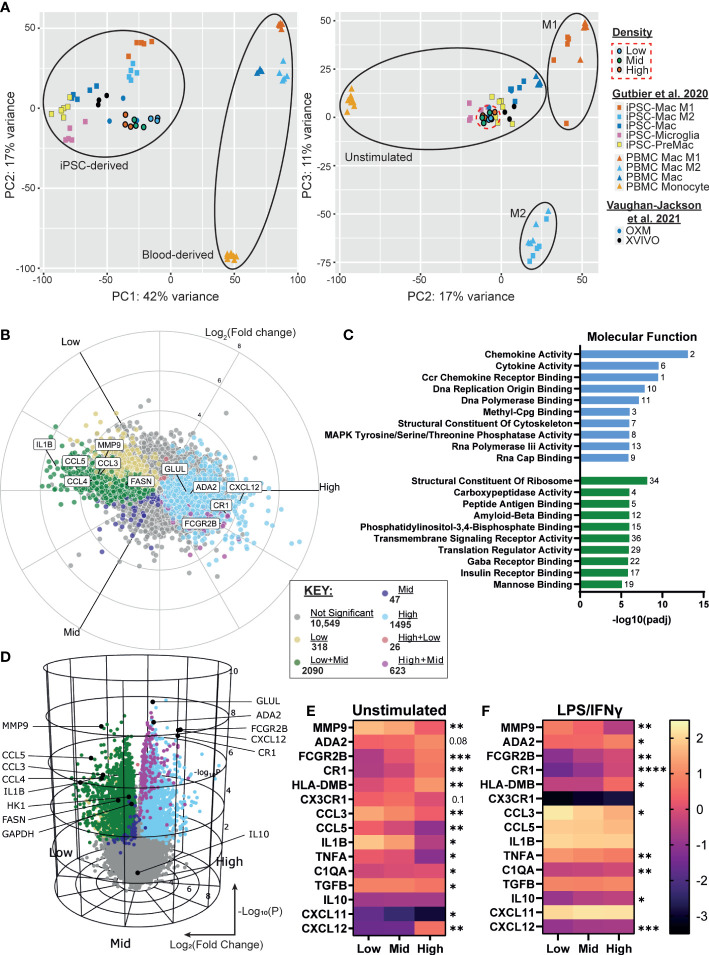
iPSC-Macs show differences in transcription at different plating densities, with low density driving a pro-inflammatory signature and high density favoring phagocytosis. **(A)** PCA comparison of different density iPSC-Macs (SFC840-03-03 iPSC line) compared to OXM or XVIVO-15 cultured iPSC-Mac (1.04x10^5^ cells/cm^2^) (35) and iPSC-Macs, microglia, MDM and M1 or M2 polarized iPSC-Mac and MDM (1.5x10^5^ cells/cm^2^) (43) **(B, C)** Differential gene expression between densities, x axis Log_2_(Fold change), y axis Log_10_(P value). **(D)** Molecular function GO terms enriched in high (green) and low (blue) density iPSC-Macs. **(E, F)** qPCR of top differential gene expression hits and cytokines identified in figure 1 in a 2^nd^ iPSC line (SFC841-03-01) **(E)** unstimulated, or **(F)** polarized. Scale shows Log_10_(2^-ΔCt^), significance indicated for low vs high comparison, n=4 two-way ANOVA, Tukey’s multiple comparisons test with Greenhouse-Geisser correction. Significance is defined as * <0.05, ** <0.01, ***<0.001, ****<0.0001.

We next sought to determine whether the density-dependent phenotypic differences could be explained by changes in gene expression. Using RNA-seq of resting iPSC-Macs at our three densities, we first compared the transcriptional profile against our prior report of iPSC-Macs cultured in OXM *versus* XVIVO-15™ based medium, as well as iPSC-Mac differentiation cultures reported by Gutbier et al. (35, 43). Principal component analysis showed that, irrespective of density, our iPSC-Macs cluster closely with unstimulated iPSC-Mac and iPSC-microglia from both published studies (**Figure 4A**). Despite the results so far suggesting M1 and M2-like polarization of low and high density cells respectively, they did not cluster with M1 and M2 polarized macrophages (**Figure 4A**, right). Analysis of gene expression found 4599/15148 genes were differentially expressed between densities. Of these, 318 were upregulated only in low density cells, and 1495 upregulated only in high (**Figures 4B, C**). Mid density cells displayed an intermediate transcriptional phenotype. 2090 genes were upregulated in both low and mid compared to high, and 623 were upregulated in both mid and high compared to low. These differentially expressed genes include cytokines such as *IL1B*, *CCL3*, *CCL4*, *CCL5* (higher in low density), and *CXCL12* (higher in high density), the expression of which was consistent with secretion analysis (**Figures 1F, G**, **4B, C**. **Supplementary File**). Gene Ontology (GO) term analysis was also consistent with our findings so far, with “chemokine activity”, “Ccr chemokine receptor binding” and “Cytokine activity” the most highly enriched terms in low density *versus* high density cells (**Figure 4D**, **S4A**). “Structural constituent of the cytoskeleton” was also enriched, consistent with **figure 2**. High density cells were enriched for receptor binding and signaling-related terms including “Peptide antigen binding” and “Transmembrane Signaling Receptor Activity” as well specific receptor binding such as insulin and mannose, consistent with increased CD206 expression (**Figure 3**). Of note, the “Major histocompatibility (MHC) class II Protein Complex” was significantly enriched (**Figure S4B**), with all constituent genes of this term robustly upregulated in high density cells (**Figure S4C**). Curiously many TLRs are more highly expressed in high density cells, ruling out density regulation of the inflammatory response being caused by increased surface sensitivity to stimulation (**Figure S4D**). Closer analysis of known TLR intracellular signaling genes indicates a more nuanced control, though. Intermediates *MyD88*, *IRAK1/2* and *TRAF6* are more highly expressed in low density macrophages. Conversely, known *TLR/NF-κB* inhibitory genes such as *SOCS3*, *PTPN6*, *TNFAIP3* and *NFKBID* are also more highly expressed at low density, as well as anti-inflammatory transcriptional regulators like *ATF3* and *NR4A2* (57) (**Figure S4E**). Why, then, with so many negative regulators of TLR signaling upregulated in low density macrophages do we see a stronger pro-inflammatory response from these cells is unclear based on transcriptomic data alone.

We confirmed our RNA-seq results by qPCR for a selection of inflammation regulating secretory and surface proteins among the top 20 differentially expressed genes across densities and identified in **Figure 1**, using macrophages from an independent genetic background (**Figure 4E**, **Supplementary Table 2**). Many of these differences remained even after LPS/IFNγ stimulation (**Figure 4F**).

Considering the higher expression of scavenger receptors and enrichment for endocytic GO terms (**Figure S4B**), we considered whether high density cells may have increased capacity for phagocytosis. Uptake of fluorophore conjugated zymosan appeared to be higher in high density cells *versus* low within 1 hour of exposure, but after 4 hours was not significantly different across densities (**Figure S4F**). Therefore, phagocytic rate, but not total capacity, appears to differ between densities. However, caution must be taken as we cannot rule out the possibility that this is due to physical limitations of the assay (*e.g.* distance between particle and cell) rather than phenotype.

### 3.5 Macrophage density is linked to altered metabolism and the extracellular environment may suppress inflammation

**Figure 5:**
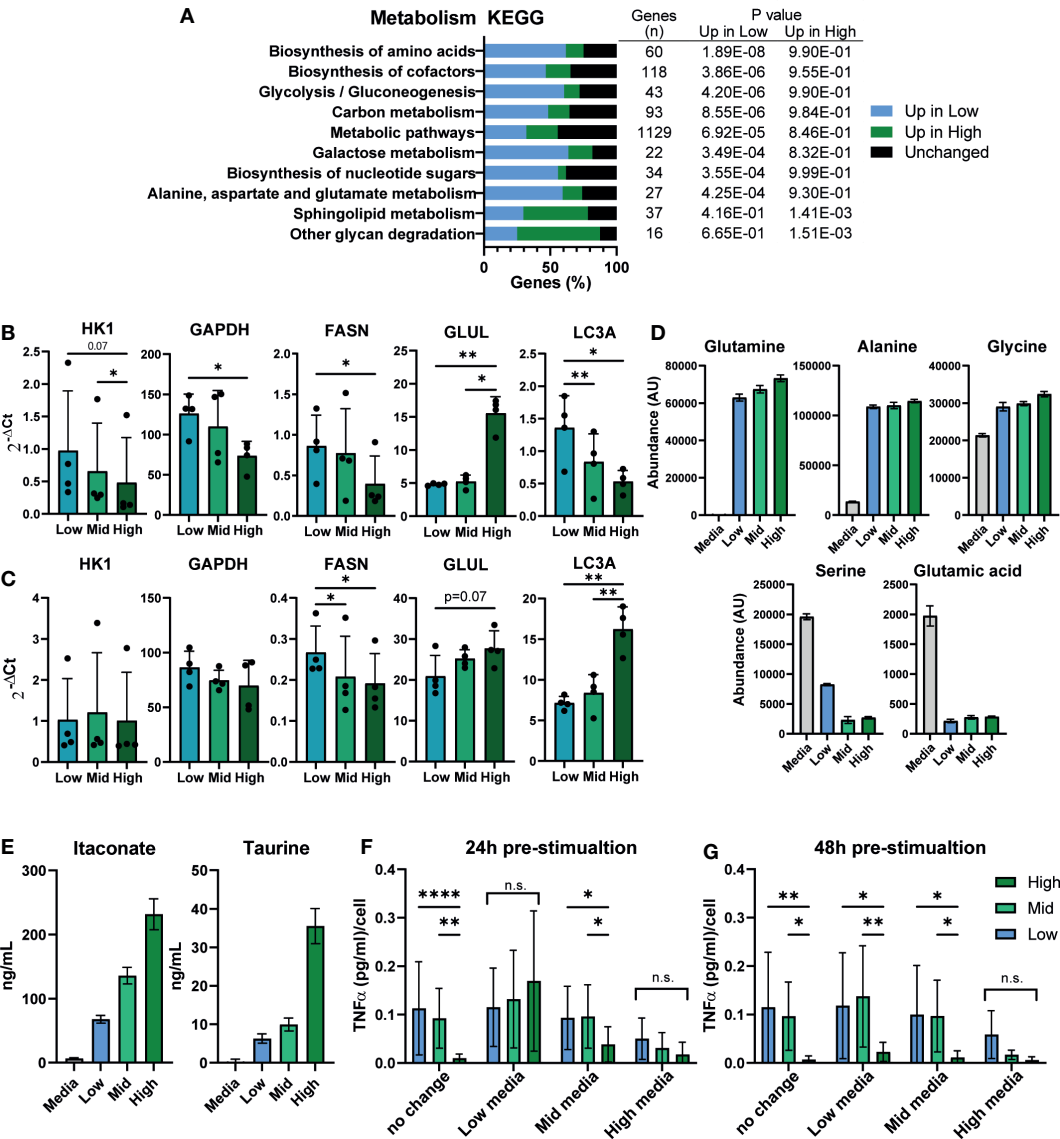
Macrophage density is linked to altered metabolism and the extracellular environment may suppress inflammation. **(A)** Metabolism related KEGG term analysis of differential gene expression comparing high vs low density plated iPSC-Mac. Bars indicate proportion of genes in a given term that are upregulated at each density or unaffected. Number of genes per term, and P value for enrichment at each density are shown in the adjoining table. **(B, C)** qPCR for metabolism related genes in a second iPSC line **(B)** unstimulated or **(C)** LPS/IFNγ polarized likely indicates broad metabolic differences between densities. n=4, significance for all genes calculated simultaneously by two-way ANOVA, Tukey’s multiple comparisons test with Greenhouse-Geisser correction. **(D)** Comparison of extracellular amino acid concentrations measured by LC-MS show limited differences between densities compared to a fresh medium control. Abundance has been calculated by area under the curve of the total ion chromatogram (TIC) peaks. **(E)** Concentration of the two immunosuppressive metabolites itaconate and taurine in the supernatant. **(D, E)** n=6 technical replicates. **(F, G)** Supernatant exchange between densities suggests a key role in regulating the density dependent inflammatory response. Media exchanged between densities **(F)** 24 hours pre-stimulation (day 6 of differentiation) or **(G)** 48 hours pre-stimulation (day 5) with 1 ng/mL LPS, and quantification of secreted TNFα 24 hours later, normalized to plated cell number. n=6, 2 iPSC lines, two-way ANOVA with Tukey’s multiple comparisons test. Significance is defined as * <0.05, ** <0.01, ***<0.001, ****<0.0001.

Macrophage inflammatory state is known to influence and to be influenced by their metabolic state, and secretion of metabolic intermediates can regulate the inflammatory response (58, 59). We investigated whether differences in macrophage density were linked to changes in iPSC-Mac metabolism. Kyoto Encyclopedia of Genes and Genomes (KEGG) term analysis of our RNA-Seq data showed enrichment of metabolic pathways, with over half of genes expressed in macrophages classified under this term differentially expressed between densities (627/1129 genes) (**Figure 5A**). Of the metabolism related KEGG terms, the most significantly enriched terms were “biosynthesis of amino acids”, “biosynthesis of cofactors”, and “Glycolysis/Gluconeogenesis”. In all 3 of these terms, more genes were upregulated in low density iPSC-Mac than high. To see how these may change after LPS/IFNγ stimulation, we selected common metabolic hallmark genes related to glycolysis (*HK1* and *GAPDH*), fatty acid synthesis (*FASN*), glutamine synthesis (*GLUL*), and autophagy (*LC3A*) to quantify by qPCR (**Figures 5B, C**). Under resting conditions, low density cells have higher expression of all genes except *GLUL*, consistent with reported metabolism of inflammatory macrophages (60, 61) (**Figure 5B**). Upon stimulation with LPS/IFNγ, *HK1* and *GAPDH* expression were no longer significantly different across densities, while *LC3A* expression became significantly higher in high density cells (**Figure 5C**). Our differentiation medium does not contain all amino acids used in protein synthesis so iPSC-Mac must synthesize their own. Also, autophagy is used during nutrient deprivation as a method of recycling intracellular components (62). Considering these facts, we asked whether the increased expression of metabolic genes, particularly those involved in amino acid biosynthesis, were due to nutrient deprivation. We used liquid chromatography–mass spectrometry (LC-MS) to determine abundance of extracellular metabolites at day 7 of iPSC-Mac terminal differentiation. The majority of extracellular amino acids showed no clear differences in abundance between different plating densities (**Supplementary Table 3**). Glutamine, alanine, and glycine abundance had increased relative to the base medium, while serine and glutamic acid were depleted across all densities (**Figure 5D**). With the possible exception of serine, it appears that amino acid availability will not explain differences in iPSC-Mac response. Furthermore, culturing macrophages in an excess volume of media did not reverse the inflammatory phenotypes observed, either at resting state, determined by qPCR of differentially expressed genes *FCGR2B*, *CXCL12*, and *IL-1β*, or after Pam3CSK4 stimulation, as shown by TNFα secretion (**Figures S5A, B**). It is, therefore, likely changes in metabolic gene expression play a more specific role in regulating these macrophages than simply as part of a starvation response. We also measured altered metabolite abundances and metabolic intermediates (**Supplementary Table 4**). Among these were several known markers and modulators of inflammation. Neopterin (63), was more abundant at low densities, and the anti-inflammatory molecules glutathione, itaconate, and the amino acid taurine (64–67), were more abundant in high density conditioned medium. Quantification of itaconate and taurine indeed demonstrated nanogram concentrations of these metabolites, 67.7 – 231.5 ng/mL and 6.3 – 35.5 ng/mL respectively (**Figure 5E**).

This would suggest that iPSC-Macs cultured at different densities may occupy different metabolite environments at the time of stimulation, which in turn may feedback on their response. To test this, we exchanged media between iPSC-Macs cultured at our different densities either 24 or 48 hours prior to stimulation with LPS (**Figures 5F, G**). When exchanged 24 hours prior to stimulation, density dependent suppression of TNFα secretion was no longer observed in either cells cultured in low or high density conditioned medium (**Figure 5F**). In low density conditioned medium, high density cells secreted more TNFα per cell, whereas low and mid density cells in high density conditioned medium produced less. Interestingly, this same effect was only seen in high density conditioned medium when media was exchanged 48 hours prior to stimulation (**Figure 5G**). This would suggest that high density cells are either actively depleting a pro-inflammatory molecule, or producing a stable anti-inflammatory molecule. While metabolites are one possible mediator of these effects, the most common and potent anti-inflammatory agent produced by macrophages is the cytokine IL-10 (68). As we have already observed that secretion of IL-10 post-stimulation appears to be unaffected by plating density (**Figure 1G**), we tested whether neutralization of extracellular IL-10 would nullify the density effect. Addition of recombinant IL-10 clearly suppressed the immune response, and this was reversed by addition of an anti-IL-10 neutralizing antibody. However, antibody treatment alone did not specifically increase TNFα secretion by high density cells compared to low density (**Figure S5C**). We therefore conclude that IL-10 is not responsible for the suppressed inflammatory response of high density macrophages. Alternatively, recent publications have identified nitrous oxide (NO) as an intermediate released into the supernatant that suppresses the macrophage inflammatory response in a density-dependent manner much like bacterial quorum sensing (26). However, inhibition of NO synthesis by L-NIL, an inhibitor of iNOS, had no effect on our iPSC-Macs (**Figure S5D**). Overall, we show here a link between density and macrophage metabolic activity. The culture supernatant, which contains varying, density dependent, concentrations of extracellular metabolites, actively enhanced or suppressed their ability to secrete inflammatory cytokines. However, as we did not remove protein components from the supernatant, we cannot rule out a potential dominant role of extracellular proteins other than IL-10 at this time.

### 3.6 Density dependent differences in phenotype are observable in MDMs and THP-1

**Figure 6:**
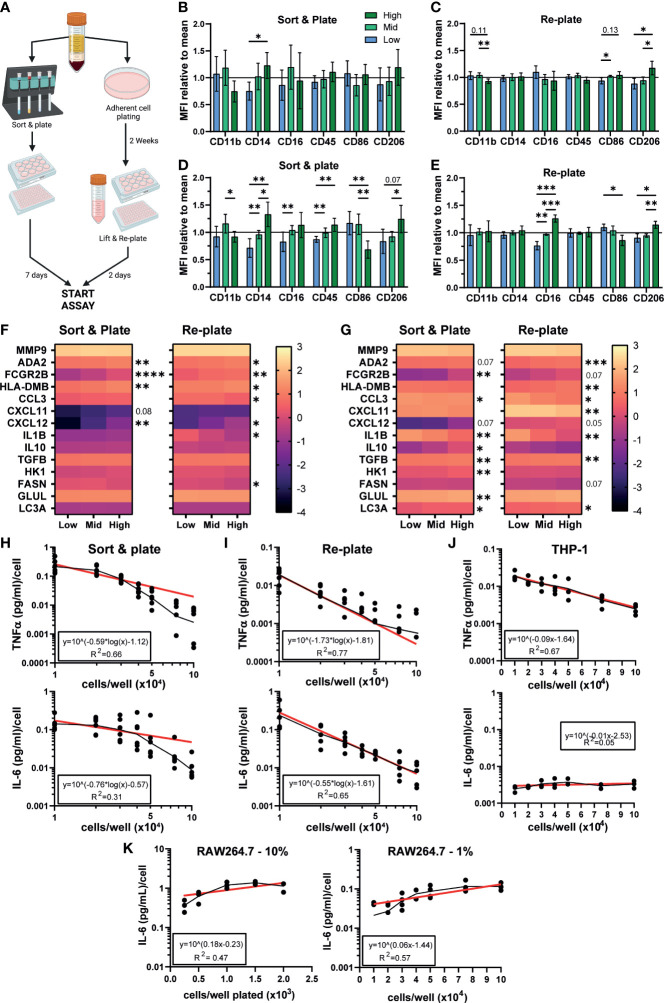
Density dependent differences in phenotype are observable in MDMs, THP-1, and iPSC-derived microglia. **(A)** Schematic of MDM isolation and plating strategies. Created with BioRender.com. **(B–E)** MDM surface marker phenotype is influenced by density. **(B, C)** MFI normalized to the mean of all 3 densities of macrophage markers on unstimulated MDM cultured *via* (left) sorting and plating the cells, or (right) replating after 2 weeks differentiation. **(D, E)** MFI normalized to the group mean of LPS/IFNγ polarized MDM. **(B–E)** “Sort & plate” n=8 independent donors, “Re-plate” n=7, Two-way ANOVA with Tukey’s multiple comparisons test and Greenhouse-Geisser correction. **(F, G)** qPCR for genes differentially expressed in iPSC-Macs at different densities in **(F)** unstimulated or **(G)** LPS/IFNγ polarized MDM. Scale represents Log_10_(2^-ΔCt^) and significance is indicated for low vs high comparison. **(F)** n=6 each for “Sort & plate” and “Re-plate”, **(G)** n=6 “Sort & plate”, n=5 “Re-plate”. **(F, G)** Two-way ANOVA, Tukey’s multiple comparisons test with Greenhouse-Geisser correction. **(H–K)** Density effect on cytokine secretion by MDM and immortalized cell lines. **(H-J)** Secretion of (top) TNFα or (bottom) IL-6, by **(H)** “Sort & plate” MDM, **(I)** “Re-plate” MDM, or **(J)** PMA-differentiated THP-1 macrophages after stimulation with 1 µg/mL Pam3CSK4. Secretion is normalized to number of cells plated. Geometric mean (black line) and Non-linear curve fit lit (red) are displayed. **(H, I)** “Re-plate” TNFα n=8, All other MDM n=6. **(J)** TNFα n=4, IL-6 n=3. **(K)** Murine RAW264.7 cultured in standard serum (10%, left, n=3) or reduced serum (1%, right, n=4) conditions, IL-6 secretion post stimulation with Pam3CSK4. Significance is defined as * <0.05, ** <0.01, ***<0.001, ****<0.0001.

We have so far demonstrated a significant effect of density on iPSC-Macs but no other macrophage models. We next tested whether primary MDMs are also influenced. Isolation methods can influence MDMs phenotype and response (69), so we isolated monocytes *via* two different approaches (**Figure 6A**). The first was by magnetic bead negative selection of monocytes followed by culturing for 7 days before stimulation, similar to our iPSC-Mac differentiation method (referred to as “Sort and plate”). Alternatively, MDM were selected by adherence to plastic. As this method gives non-exact plating densities, these cells were allowed to conclusively differentiate for 2 weeks. Subsequently, cells were lifted and re-plated at desired densities for another 48 hours before stimulation (here referred to as “Re-plate”). Morphological differences like those seen with iPSC-Macs were not always observed in MDM due to donor variability (**Figure S6A**). However, surface marker expression did display density dependent changes (**Figures 6B, C**, **S6B, C**). In “Sort & plate” MDM, lower expression of CD14 was observed in low density cells while “Re-plate” MDM showed significant differences in CD11b (low > high), CD86 and CD206 (high > low), but not CD14. After stimulation with LPS/IFNγ, “Sort & plate” MDM show very similar trends as iPSC-Macs, with reduced CD14, CD16, CD45, and CD206 in low density cells relative to high, and increased CD86 (**Figures 6D**, **S6D, E**). “Re-plate” MDM did not mirror iPSC-Macs, but none-the-less had the same pattern of CD16, CD86, and CD206 expression (**Figures 6E**, **S6F, G**). Transcriptional analysis by qPCR for the secretory, surface, and metabolic genes measured in iPSC-Macs (**Figures 4E**, **5B**), again showed differences between densities (**Figures 6F, G**). Unstimulated “Sort & plate” and “Re-plate” MDM had higher expression of *ADA2*, *FCGR2B*, *HLA-DMB*, and *CXCL12* in high density cells. In addition, “Re-plate” low density cells had higher expression of *CCL3*, *IL1B*, and *FASN* (**Figure 6F**). After stimulation with LPS/IFNγ, gene expression patterns in MDM were largely similar to those in iPSC-Macs, with higher expression of cytokines and chemokines at low density, and higher expression of surface markers as well *GLUL* and *LC3A* at high (**Figure 6G**). However, some features were unique to MDM, namely higher expression of the anti-inflammatory molecule *TGFB*, and of the glycolysis related *HK1* in “Sort & plate” MDM. Finally, secretion of TNFα and IL-6 by MDM after TLR2 stimulation reflected iPSC-Macs, declining significantly as cell density increased regardless of isolation method (**Figures 6H, I**, **1B**, **S1D**). Therefore, MDM also display density dependent regulation of their inflammatory state consistent with that observed in iPSC-mac, with some variation depending on isolation method used.

We next examined whether the human monocytic THP-1 immortalized cell line showed density dependent inflammatory features. Curiously, while TNFα secretion per cell declined as density increased after TLR2 stimulation, IL-6 secretion did not (**Figure 6J**). This IL-6 pattern was also seen with the mouse peritoneal macrophage-like RAW264.7 cell line, both cultured in standard (10%) serum conditions or low (1%) serum conditions to slow down cell replication (**Figure 6K**). We conclude that this density dependent homeostatic function displayed by authentic macrophages is not-well modelled by immortalized cell lines.

### 3.7 Density influences in vitro modeling of neurodegenerative diseases

**Figure 7:**
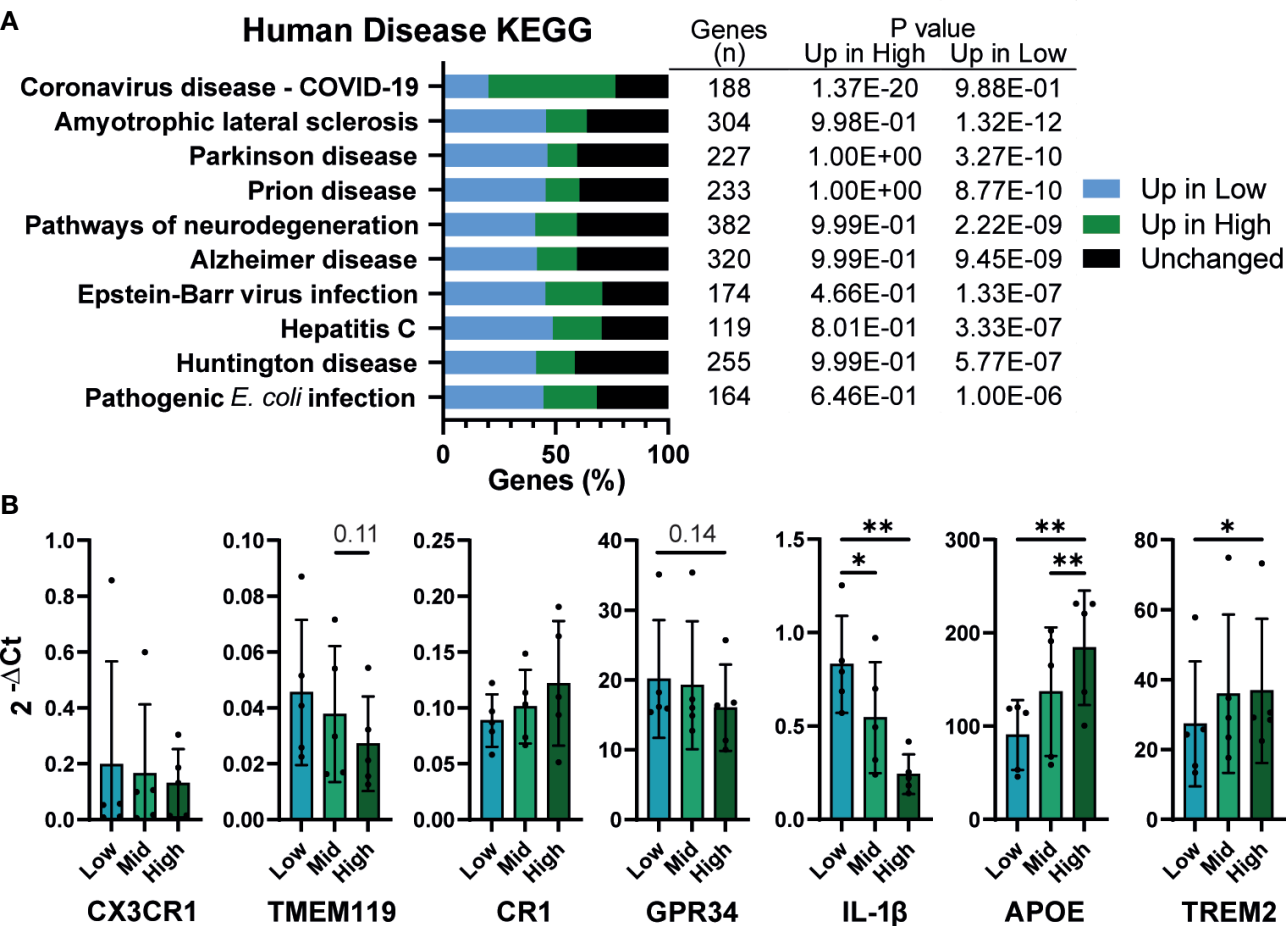
Density influences *in vitro* modeling of neurodegenerative diseases **(A)** Human disease related KEGG term analysis of differential gene expression comparing high *vs* low density plated iPSC-Mac. Bars indicate proportion of genes in a given term that are upregulated at each density or unaffected. Number of genes per term, and P value for enrichment at each density are shown in the adjoining table. **(B)** iPSC-derived microglia differentiation is influenced by density. qPCR for microglial genes and genes related to Alzheimer’s disease. n=5, Two-way ANOVA, Tukey’s multiple comparisons test, with Greenhouse-Geisser correction. Significance is defined as * <0.05, ** <0.01.

Finally, KEGG analysis for pathways associated with human disease were, unsurprisingly, enriched for inflammatory diseases. However, strikingly, half of the top 10 enriched terms were related to neurodegenerative diseases, including Amyotrophic lateral sclerosis (ALS), Parkinson’s, Alzheimer’s, and Huntington’s disease (**Figure 7A**). iPSC derived microglia (iMG) have emerged as a powerful tool for studying neurodegeneration, with numerous protocols described for their derivation *in vitro* (70, 71). Given that these protocols use a wide range of plating densities for generating microglia, (~2x10^4^ – 1x10^5^/cm^2^), it is important to determine whether density dependent changes in phenotype are also apparent in iMG. Low density iMG had improved morphology compared to high (**Figure S7**) and fewer cells were lost during differentiation. The typical genetic markers of microglia *CX3CR1*, *TMEM119*, *CR1 and GPR34* were not significantly affected by density (**Figure 7B**). However, 2 genes identified by genome wide association studies (GWAS) for Alzheimer’s disease, *APOE* and *TREM2* (72), were upregulated at high density. Consistent with our iPSC-Mac results, *IL-1β* transcription was increased in low density microglia. In conclusion, macrophage density influences microglia differentiation and may be relevant to the breakdown of homeostasis in neurodegenerative disease.

## 4 Discussion

We here describe a feedback system whereby increasing macrophage plating density decreased their ability to produce cytokines and chemokines in response to pro-inflammatory stimulation. This density effect was accompanied by clear differences in morphology, surface marker expression, and transcriptional profile before and after stimulation. These observations were not unique to iPSC-derived cells but could also be seen in MDM, and to some extent THP-1-derived macrophages. This system is distinct from recent similar reports (26, 30, 31), highlighting the multifaceted means by which density feeds back to suppress inflammation.

Changes in cytokine production by macrophages were irrespective of cell death or total expression of the receptors that initiate these responses. Therefore, it is likely that density regulates intracellular signals to influence the inflammatory response. Of the secretory proteins measured, only the chemokines CXCL11, CXCL12, and the anti-inflammatory cytokine IL-10 were not significantly influenced by density (73, 74). While secretion of CXCL12 was largely similar between resting and classically activated iPSC-Mac, and is therefore likely independent of the pro-inflammatory response, CXCL11 secretion increased over 5-fold after LPS/IFNγ stimulation. As well as binding to its primary receptors CXCR3 and CXCR7, CXCL11 also competitively binds CCR3 and CCR5 to antagonize CCL3, CCL4, and CCL5 activity (75). All three of these were upregulated in low-density macrophages, so, in this context, CXCL11 may act as an anti-inflammatory molecule alongside IL-10. This still leaves the importance of CXCL12 unclear. However, given changes in CXCL12 were conserved across cell types and states, we could leverage this to explore density dependent changes in the transcriptome. Comparative ATAC-Seq or ChIP-Seq between CXCL12, CXCL11 and IL-10 against low density upregulated cytokines like CCL3 and IL-1β could identify critical transcription factors involved. For example, metabolic transcription factors and nuclear receptors such as PPARγ and LXR are known to link inflammation and metabolism *via* “transrepression”, thus potentially also explaining differences in metabolite environment we observed (76).

It is becoming increasingly apparent the essential role metabolism and the metabolite environment play in regulating inflammation (58, 59, 61). However, the medium cells are cultured in typically has excessive concentrations of most metabolites needed for cell survival. For example, glucose concentration in our differentiation medium is approximately 17 mM compared to 4 mM in the blood, and amino acid concentrations range anywhere from 4 times lower to 10 times higher than plasma concentrations (35, 77). During 7-day differentiation these metabolites will become depleted and replaced with intermediates and byproducts. Here, we quantified itaconate and taurine as two known anti-inflammatory products (64, 66), but others that we also detected were lactate, acetoacetate and fumarate, all of which have anti-inflammatory effects (78–80). Alternatively, we also identify potentially pro-inflammatory molecules like palmitate and sialic acid (81, 82). Density appears to influence the accumulation of these metabolites. While production of each may be equal per cell across densities, they could potentially act as quorum triggers, much like ROS and other cytokines have been described to behave in other macrophage models (27, 29, 83). While we ruled out the importance of NO in our human tissue macrophages (identified by Postat and colleagues in mice), a range of metabolites and their products could be responsible for density dependent influences in our *in vitro* model. The field may need to consider revisiting current differentiation protocols, setting clearer requirements for feeding frequency, and shortening the differentiation process to reduce metabolite concentration variability.

Spatial confinement of macrophages has been reported to suppress their inflammation (31, 84). While these studies were carried out primarily in murine bone marrow-derived macrophage, the mechanism defined by Jain and Vogel was also confirmed in in RAW264.7 cells which we found did not display density-dependent regulation of IL-6 secretion. Therefore, changes specifically in HDAC3 activity they reported likely do not fully explain the density-dependent changes we have observed. Nonetheless, we cannot rule out potentially important differences in cytoskeletal shape and of structures we observed such as the tunnelling nanotube-like structures between stimulated high-density macrophages. The importance of nanotubes is unclear, but they have been reported to transfer small organelles, antigens, and even pro-phagocytic signals (53, 85). Alternatively, cytoskeletal rearrangements and physical forces are known influencers of macrophage phenotype (86–88). Changes in substrate rigidity alter the macrophage inflammatory response due to actin dependent altered localization of YAP, a transcription factor important for contact inhibition signaling (23, 24, 89, 90). Meli et al. describe this system as density-independent, and Patel et al. demonstrated elasticity influences both RAW264.7 cells as well as human alveolar macrophages, so it is unclear whether these principles apply to our iPSC-Mac results. However, remodeling of the extracellular matrix, by enzymes like matrix metalloproteinases (highly expressed by low density macrophages), and clustering of cells (seen with stimulated high density macrophages) could alter local environment rigidity both *in vitro* and *in vivo*. Therefore, exploring surface rigidity and cytoskeleton-dependent influences on density-dependent phenotype changes will need to be evaluated.

We identified increased expression, transcriptional and proteomic, of CD14, CD206, CR1 (CD35), FCGR2B (CD32), and TREM2 in high density iPSC-Mac and iMG. All of these are important for phagocytosis (91–95). Combined with enrichment of GO terms relevant to phagocytosis and our phagocytosis assay, we propose that high density cells are more phagocytic than low density macrophages. Depending on the meal, phagocytosis can either stimulate or suppress inflammation, and phagocytosis of apoptotic cells is a hallmark of resolution phase macrophages (2, 96, 97). In this regard, density-dependent changes may reflect a transition of macrophages from pro-inflammatory initiators to anti-inflammatory resolvers of the immune response. However, we also observed upregulation of peptide antigen binding and MHC class II complex proteins in high density macrophages. Macrophages, along with dendritic cells, are professional antigen presenting cells. After phagocytosis, tissue resident macrophages process antigens for presentation on MHC class II as well as cross present antigens on MHC class I for activation of T cells with the help of co-stimulatory receptors (98, 99). Therefore, an alternative possibility is that, while low density macrophages drive leukocyte recruitment, high density macrophages decide whether to activate the T-cell adaptive immune response. Future work looking at T-cell recruitment and mode of activation (*e.g.* pro-inflammatory T_h_1 or anti-inflammatory T_h_2) by different density macrophages will, therefore, be of great interest.

The most important question now is whether density-dependent features identified here reflect *in vivo* maintenance of macrophage distribution density, homeostasis, and immune responses. Cytokine, chemokine, and metabolite concentrations all change locally *in vivo* during inflammation. As too does the extracellular matrix composition and rigidity, and the presence of apoptotic and necroptotic cells to be phagocytosed. Therefore, all potential regulators of the density phenotypes discussed here could theoretically influence *in vivo* macrophage phenotypes and functions. On the other hand, unlike *in vitro* monocultures, the *in vivo* environment is filled with stromal cells that can act as sources or sinks of these same stimuli. Indeed, we have previously observed that *in vitro* co-culturing of microglia with neurons suppresses inflammatory cytokine production by the microglia (34). The importance of determining density dependent regulation of macrophages *in vivo* is emphasized by our identification of known Alzheimer’s related genes, *APOE* and *TREM2*, as being differentially expressed across microglia densities *in vitro* (72). Furthermore, several neurodegenerative disease KEGG pathways are enriched in our iPSC-Mac, identifying this density-dependent regulatory system, or the breakdown of it, as a potential contributor to these diseases. The results of this current study suggest then that we ought not to overlook the importance of density when attempting to model these diseases *in vitro*, at the risk of inadvertently occluding significant functional observations.

In conclusion, density dependent regulation of tissue macrophages may have substantial implications for *in vivo* macrophage homeostasis and the progression of human diseases. We contend that *in vitro* plating density is an often-overlooked important variable in macrophage modelling that should be given greater consideration in the future. However, we would like to note that we do not recommend a specific plating density at which to culture macrophages, as different densities may perform better for different given assays. Instead, we hope that the data provided here will act as a resource for others to compare against in their own research, and aid in scientific reproducibility.

## Data availability statement

The datasets presented in this study can be found in online repositories. The names of the repository/repositories and accession number(s) can be found below: https://www.ncbi.nlm.nih.gov/geo/, GSE197865.

## Author contributions

Conceptualization, AV-J. Methodology, AV-J. Investigation, AV-J, KE, EJ and SS. Data Curation, SS. Formal Analysis, AV-J, SS and KE. Visualization, AV-J, SS, KE, EJ and PR. Resources, EJ, MD, SZ and JM. Supervision, PR and JM. Writing – Original Draft, AV-J and WJ. Writing – Review and Editing, AV-J, KE, MD, JM, and WJ. Funding Acquisition, AV-J, WJ, and JM. All authors contributed to the article and approved the submitted version.
