# Supplementary material for: Density dependent regulation of inflammatory responses in macrophages
Source: Front Immunol. 2022 Dec 16;13:895488. doi: 10.3389/fimmu.2022.895488 (PMC9800520; doi:10.3389/fimmu.2022.895488)
Supplement: Supplementary file 1 [file DataSheet_1.pdf]

Supplementary Material

1. Supplementary Figures

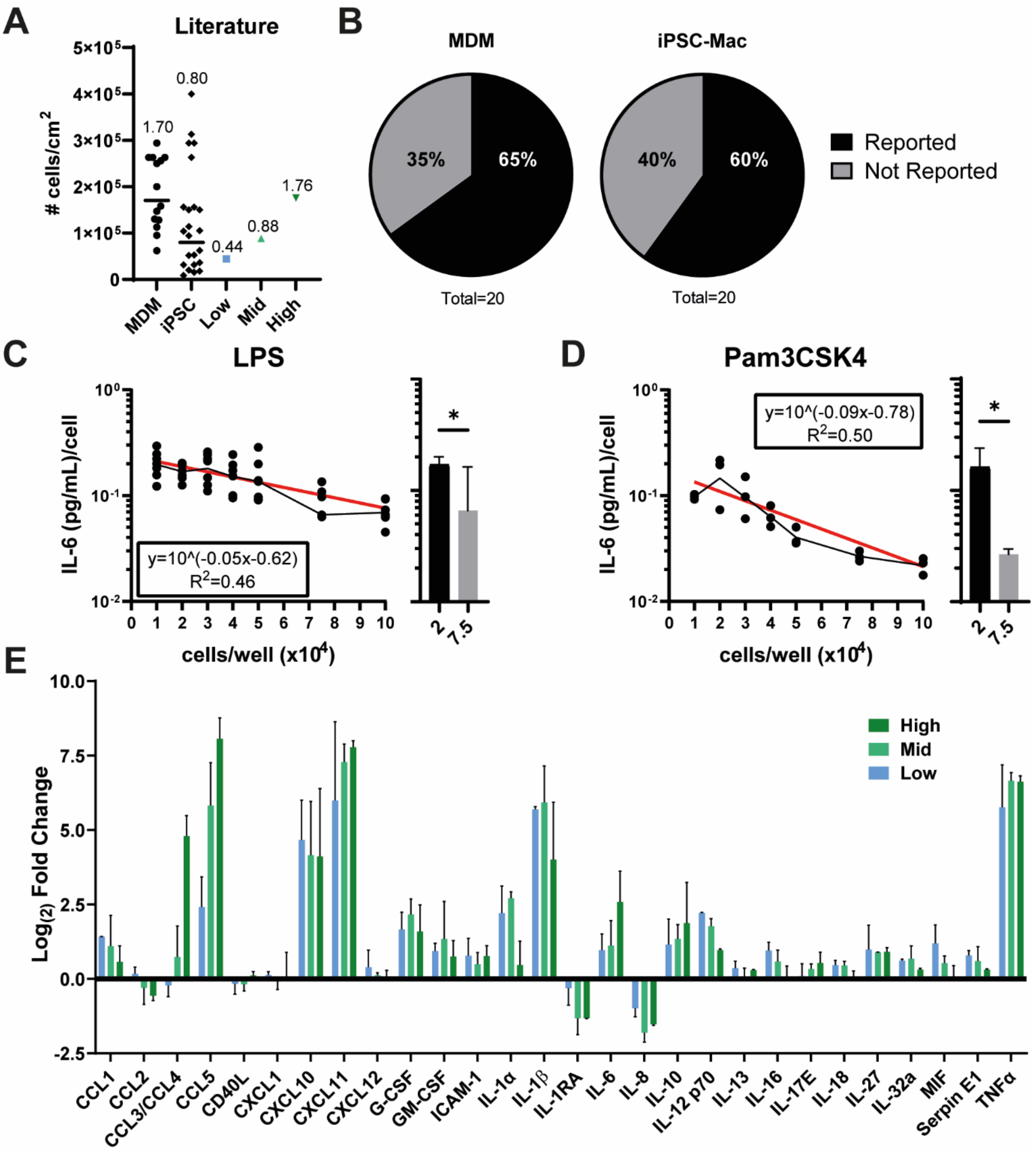

Figure S1, related to figure 1 supplementary table 1:

Plating Density is poorly reported. Analysis of 20 randomly selected papers from Scientific Reports that report using MDM published in the last 4 years, and 20 iPSC-Mac methods papers published after 2009 (see supplementary table 1 for complete list) for reporting plating density. **(A)** Scatter plot shows a broad range of plating densities utilised for either cell type. Geometric mean density of cell/cm^2 is shown above sample points. Also shown are the plating densities "low", "mid", and "high" referred to throughout this manuscript. **(B)** Within these publications, 35% of MDM manuscripts and 40% of iPSC-Mac manuscripts fail to clearly report plating density, either by omission, or reporting as cell/mL, which is meaningless for adherent cells

without knowing the volumes used. **(C-D)** IL-6 secretion from iPSC-Macs stimulated with **(C)** LPS 1 ng/mL or **(D)** Pam3CSK4 1 µg/mL for 24 hours. Concentration normalised to number of cells plated. Geometric mean (black line) and non-linear regression curve (red line) shown. Histograms show mean +/- SD. Significance calculated by pairwise comparison of $2x10^4$ vs $7.5x10^4$ cells/well, ratio paired T-test; **(C)** n=7 across 3 independent iPSC lines, **(D)** n=3, 1 iPSC line. **(E)** Log_2(Fold Change) of cytokines and chemokines shown in figure 1f-g shows upregulation of most except CCL2, CD40L, IL-1RA, and IL-8 which are downregulated. n=3. Significance is defined as * <0.05, ** <0.01, ***<0.001, ****<0.0001.

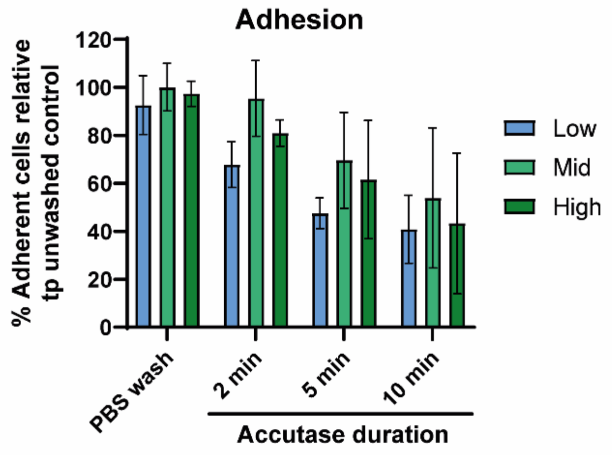

Figure S2, related to figure 2:
Adherence of macrophages determined by resistance to enzymatic lifting by Accutase™ as previously described (1). Mean +/- SD, n=4, 2 iPSC cell lines. No significant differences as calculated by Two-way ANOVA, Tukey’s multiple comparisons test with Greenhouse-Geisser correction. Significance is defined as * <0.05, ** <0.01, ***<0.001, ****<0.0001.

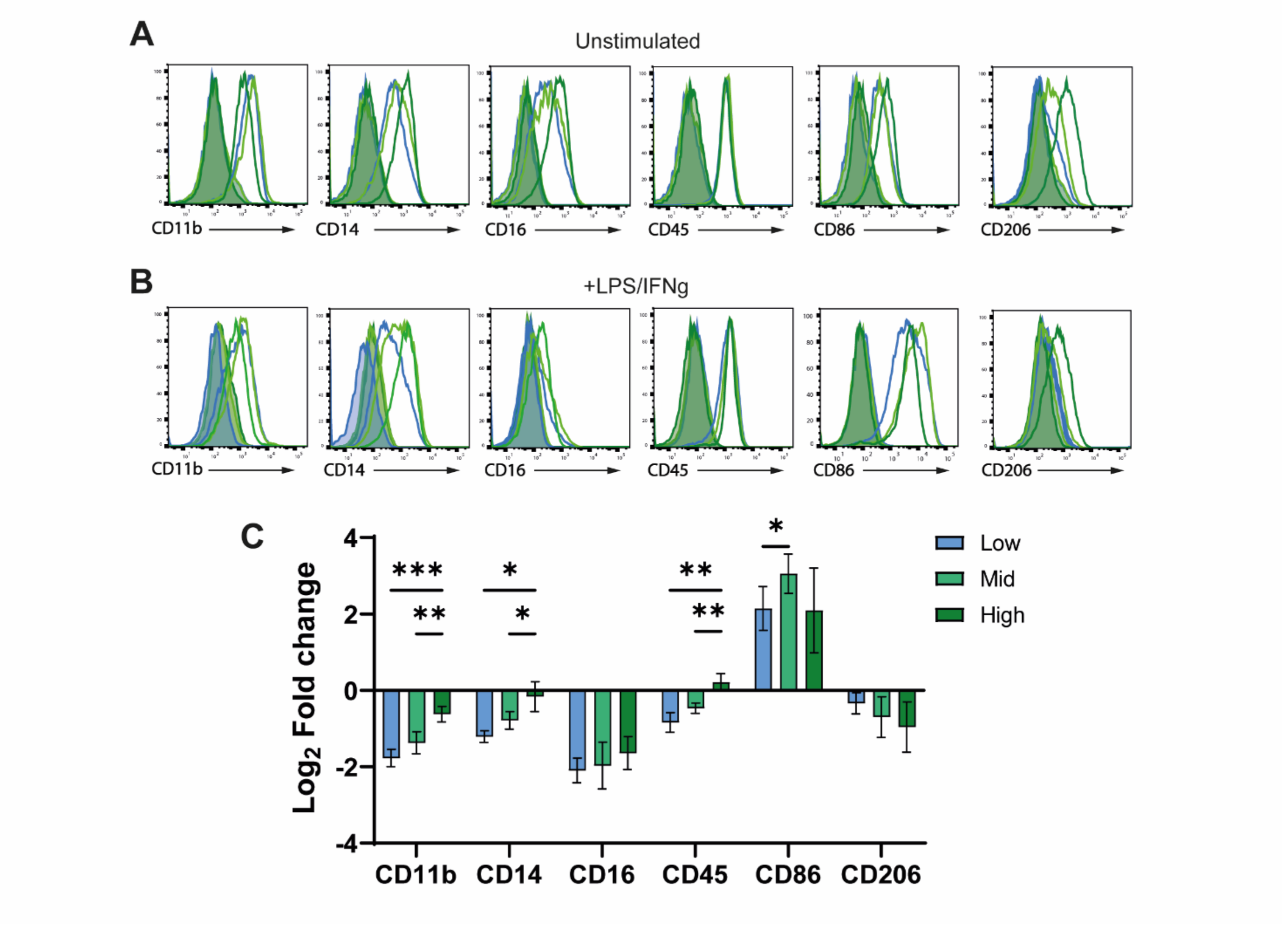

Figure S3, related to figure 3:

(A-B) Histograms representing surface marker staining (no-fill histogram) compared against the isotype control for each (solid fill histogram). **(A)** Unstimulated iPSC-Mac, **(B)** iPSC-Mac stimulated with LPS and IFNγ for 24 hours. **(C)** Log_2(Fold Change) of macrophage surface markers after the 24 hour stimulation with LPS and IFNγ shows reduction of all markers on low and mid density cells except CD86. Mean +/- SD, n=5 (CD86 n=4), 1 iPSC line, mixed effects analysis, Tukey's multiple comparisons test with Greenhouse-Geisser correction. Significance is defined as * <0.05, ** <0.01, ***<0.001, ****<0.0001.

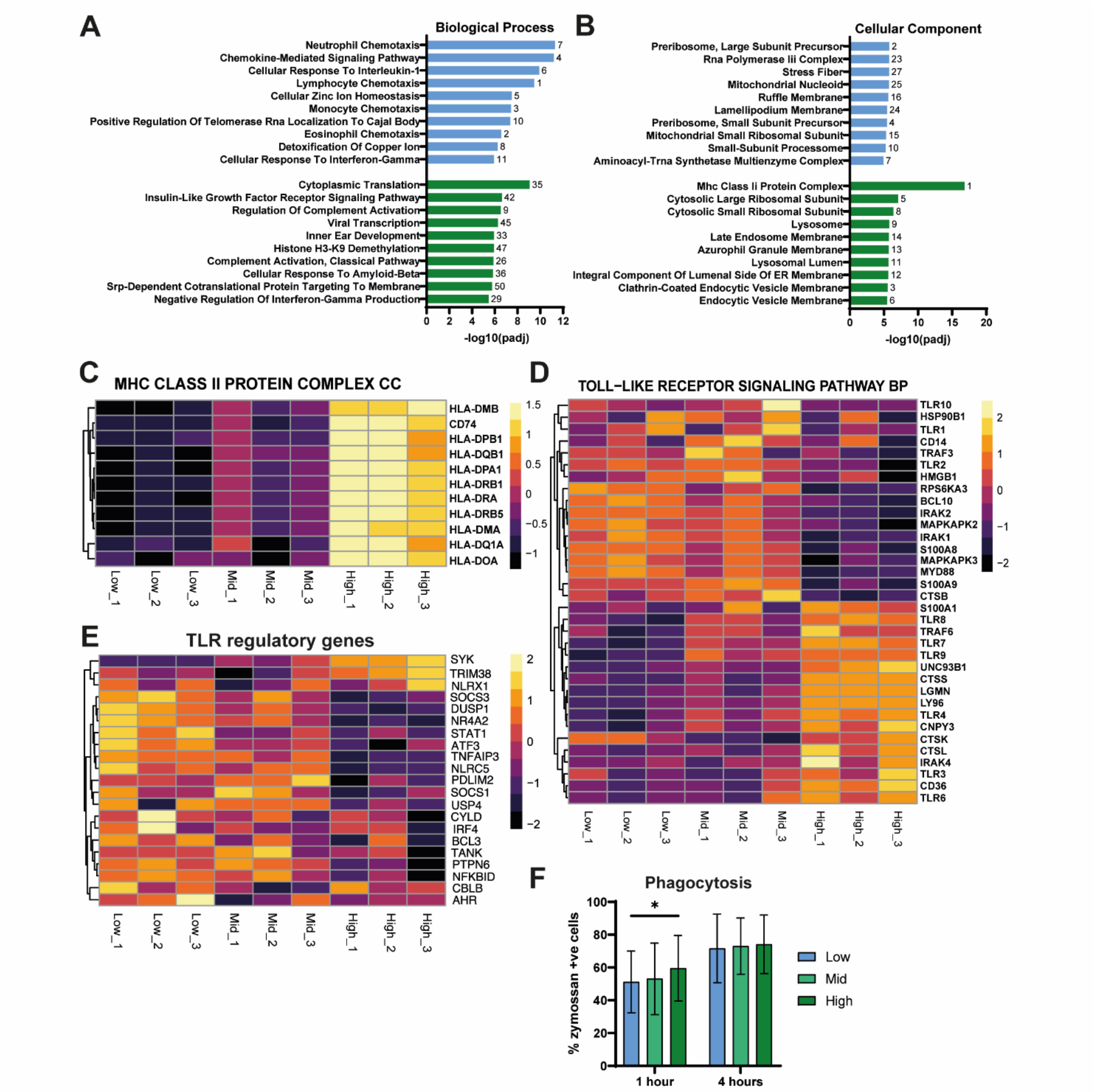

Figure S4, related to figure 4:

(A) Biological Process (BP) and **(B)** Cellular Component (CC) GO terms enriched in high (green) and low (blue) density iPSC-Macs. **(C)** Heatmaps of GO terms for; “MHC Class II protein complex CC” and **(D)** “Toll-Like receptor signaling pathway BP”. Results across 3 repeat measurements are shown. The color scale represents the z-score of the log_2 fold difference from the mean TPM value for that gene. **(E)** Heatmap of TLR-regulatory genes curated from Kondo, Kawai, & Akira (2012) (2). **(F)** Percentage of cells that have phagocytosed Zymossan-488 bioparticles™ 1 hour or 4 hours after addition. Mean +/- SD, n=6, 1 iPSC line. Significance calculated by Two-way ANOVA, Tukey’s multiple comparisons test. Significance is defined as * <0.05, ** <0.01, ***<0.001, ****<0.0001.

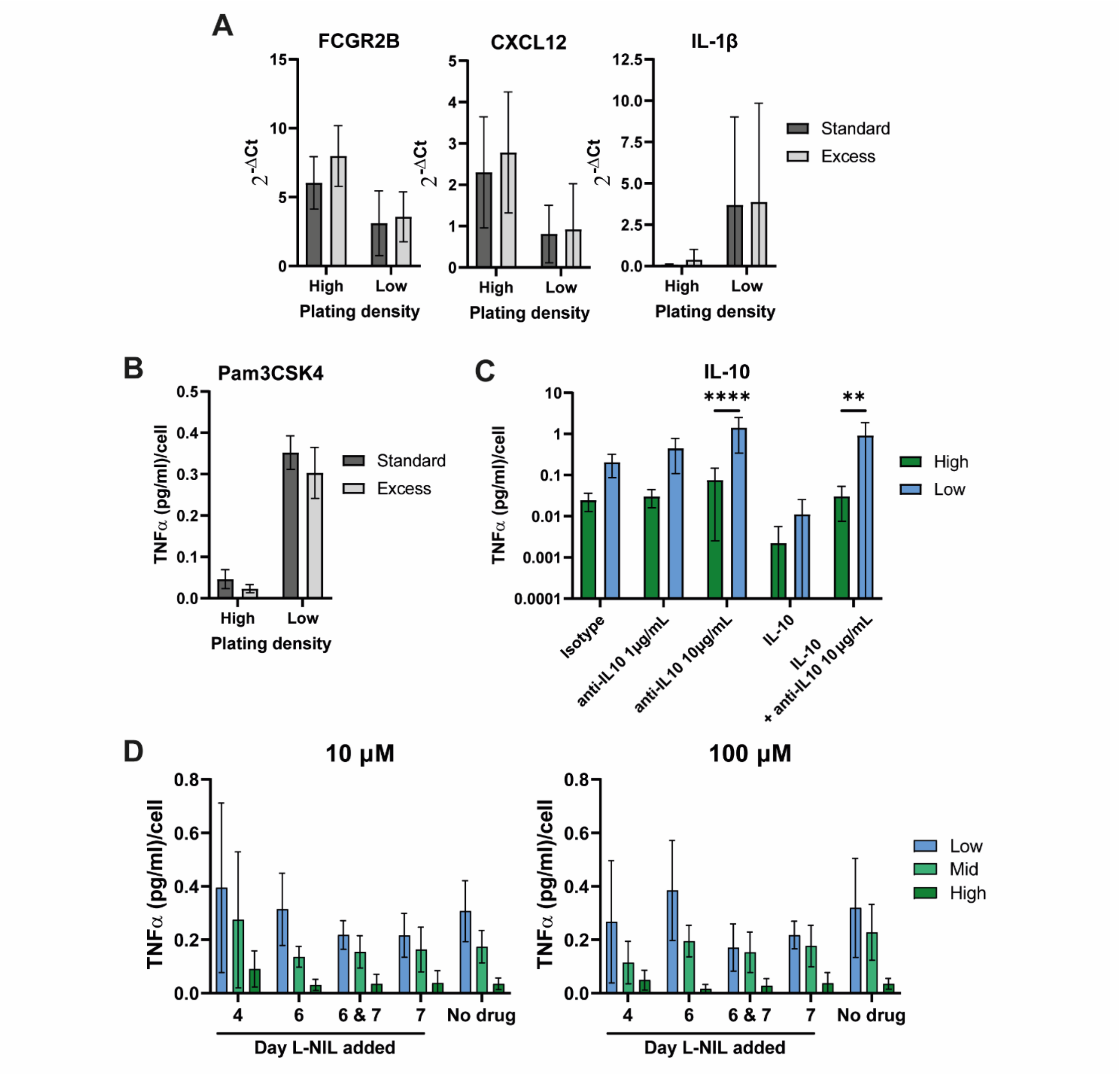

Figure S5, related to figure 5:

(A-B) Density effects are not due to nutrient depletion. **(A)** qPCR of density influenced genes in resting iPSC-Macs cultured in either a standard volume of media, or an excess (2X) volume. **(B)** TNFα secretion after a 24-hour stimulation with 1 µM Pam3CSK4 from iPSC-Macs cultured in standard or excess media volumes. **(A-B**) Mean +/- SD, n=6, 3 iPSC cell lines. **(C)** Inhibition of IL-10 does not reverse density effects on inflammation. iPSC-Macs were treated with either 10 µg isotype control antibody, 1 µg/mL or 10 µg/mL IL-10 neutralising antibody, 20 ng/mL IL-10 or both IL-10 and 10 µg neutralising antibody, 24 hours prior to stimulation with 1 µg/mL Pam3CSK4. Mean +/- SD, n=6, 3 iPSC cell lines. Two-way ANOVA, Sidaks's multiple comparisons test. Significance is defined as * <0.05, ** <0.01, ***<0.001, ****<0.0001. **(D)** NO synthesis inhibited by the addition of 10 µM or 100 µM NO inhibitor L-NIL (3) on day 4, 6, 7 or both 6 and 7, of terminal macrophage differentiation. Where added on day 7, this was in combination with the agonist. iPSC-Macs were then stimulated for 24 hours with Pam3CSK4 1 µg/mL and TNFα secretion normalised to cell plating number is shown. Mean +/- SD, n=3, 2 iPSC cell lines.

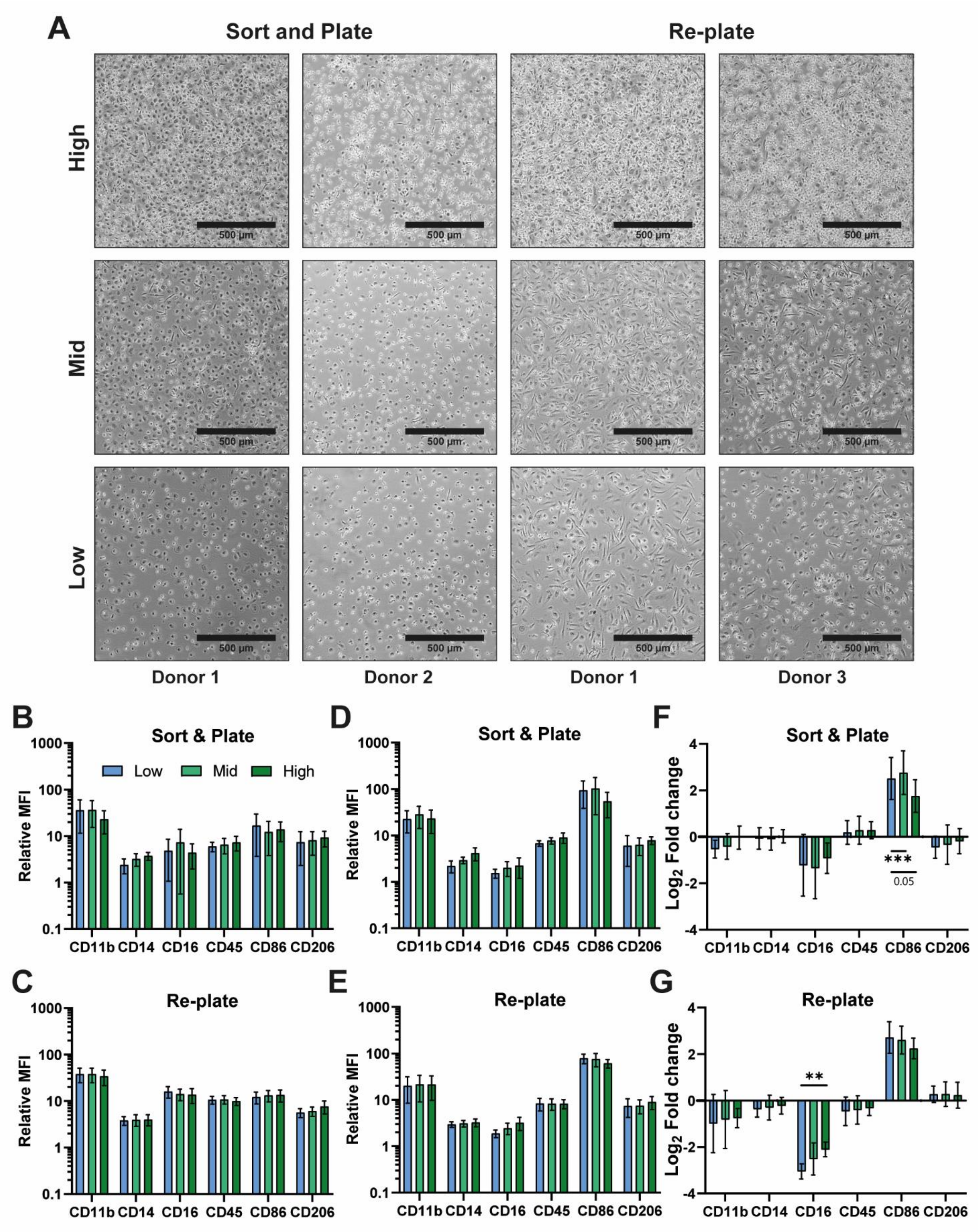

Figure S6, related to figure 6:

(A) MDM morphology varied depending on donor and plating method. **(B-E)** Un-normalized MFI for macrophage markers on MDM corresponding to figure 6b-e. **(F-G)** Log_2(Fold Change) of surface marker post LPS and IFNγ stimulation for 24 hours. **(B, D, F)** n=8, **(C, E, G)** n=7. Mean +/- SD. Two-way ANOVA, Tukey's multiple comparisons test with Greenhouse-Geisser correction. Significance is defined as * <0.05, ** <0.01, ***<0.001, ****<0.0001.

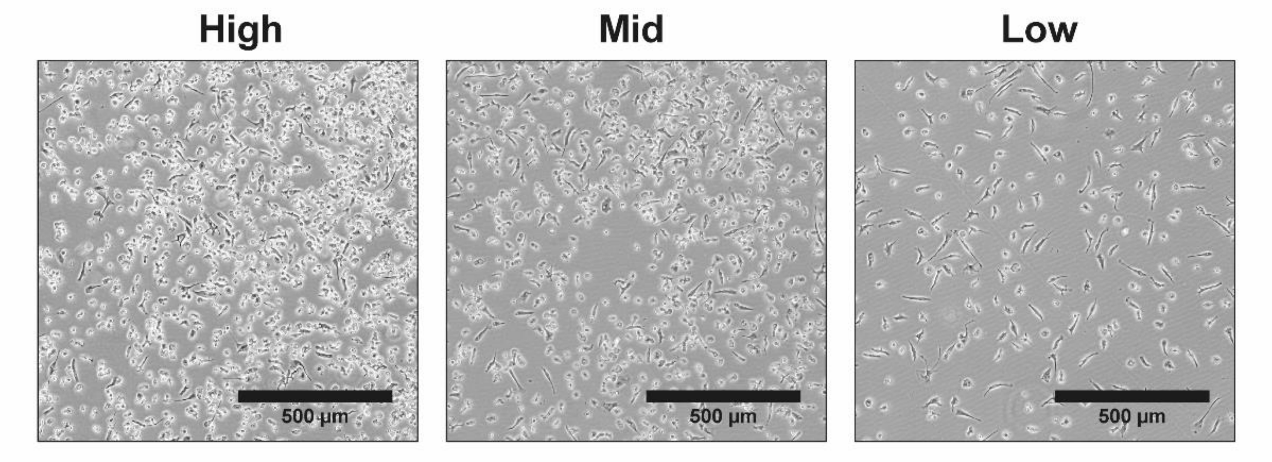

Figure S7, related to figure 7:
Morphology of iPSC-microglia after the 14-day differentiation method (4). Images representative of 4 repeats.

2. Supplementary Tables:

Cell type	Final differentiation duration (days)	Frequency of Feeding (days)	Plating Density (/cm^2)	Reference
MDM	6	0	N/A	(5)
MDM	7	0	2.50E+05	(6)
MDM	5-11	0	N/A	(7)
MDM	5	0	2.56E+05	(8)
MDM	7	0	N/A	(9)
MDM	14	7	1.13E+05	(10)
MDM	5	0	1.30E+05	(11)
MDM	6	0	N/A	(12)
MDM	7	0	2.63E+05	(13)
MDM	7	2	N/A	(14)
MDM	7	0	1.28E+05	(15)
MDM	6-7	3	2.63E+05	(16)
MDM	6	0	2.94E+05	(17)
MDM	7	2	2.00E+05	(18)
MDM	6	0	1.58E+05	(19)
MDM	7	0	2.63E+05	(20)
MDM	6	0	6.20E+04	(21)
MDM	5-7	0	N/A	(22)
MDM	7	0	1.47E+05	(23)
MDM	7-10	0	N/A	(24)
iPSC-Mac	5-7	3	N/A	(25)
iPSC-Mac	10-14	0	1.8-3.6E+04	(26)
iPSC-Mac	5-7	3	2.00E+04	(27)
iPSC-Mac	4	0	N/A	(28)
iPSC-Mac	14	7	N/A	(29)
iPSC-Mac	3-7	0	2.94E+05	(30)
iPSC-Mac	5-7	0	1.56E+05	(31)
iPSC-Mac	7	4	1.56E+05	(32)
iPSC-Mac	7	3-4	N/A	(33)
iPSC-Mac	7-10	0	N/A	(34)
iPSC-Mac	5-10	0	8.82E+03	(35)
iPSC-Mac	9	3	1.04E+05	(36)
iPSC-Mac	7	0	2.63E+05	(37)
iPSC-Mac	3	0	N/A	(38)
iPSC-Mac	6	0	1.56E+04	(39)
iPSC-Mac	7	0	N/A	(40)
iPSC-Mac	4	0	4.00E+05	(41)
iPSC-Mac	7	3-4	1.50E+05	(42)
iPSC-Mac	7	0	N/A	(43)
iPSC-Mac	7	4	1.05E+05	(1)

Supplementary Table 1, relating to the introduction and figure S1: Literature analysis for plating density

Plating density of human MDM taken from 20 randomly selected publications in the journal Scientific Reports published within the last 4 years. Plating density of human iPSC-Mac are from methodology publications published since 2009 and previously identified (1). Frequency of feeding is shown were available. A value of 0 either means cells are not fed throughout final differentiation, or frequency is not reported. Where plating density has either not been reported, or been given as cell/mL (meaningless for adherent cells without also reporting volume used) density is shown as N/A.

High vs Low			High vs Mid			Mid vs Low		
**Gene**	**log_2FC**	**adj.P.Val**	**Gene**	**log_2FC**	**adj.P.Val**	**Gene**	**log_2FC**	**adj.P.Val**
MMP9	-3.545	3.72E-07	MMP9	-2.726	2.59E-06	TNC	-2.273	4.96E-04
PIK3IP1	2.822	6.57E-07	PIK3IP1	2.439	2.59E-06	FCGR2B	1.894	1.26E-03
CR1	4.102	6.57E-07	SLC18B1	2.587	5.78E-06	PYGL	1.092	1.26E-03
FCGR2B	4.180	6.57E-07	ABCC5	1.687	5.78E-06	TNFSF14	-2.648	1.26E-03
SLC18B1	3.007	1.19E-06	CXCL12	3.593	5.78E-06	CR1	1.403	4.54E-03
CXCL8	-4.195	1.19E-06	CR1	2.699	6.30E-06	LILRB5	1.657	4.54E-03
C15orf48	-2.642	1.19E-06	GPNMB	2.456	6.30E-06	MCOLN3	-1.177	4.54E-03
GPNMB	2.891	1.19E-06	NCF1C	-2.914	6.30E-06	MARCKSL1	-1.437	4.54E-03
MS4A4A	3.339	1.19E-06	NCF1	-3.093	6.56E-06	NA	1.372	5.98E-03
HLA-DMB	4.174	1.19E-06	SLC2A6	-2.509	6.30E-06	MMP9	-0.819	5.98E-03
ABCC5	1.817	1.19E-06	FCGR2B	2.286	7.20E-06	MYO7A	1.467	5.98E-03
SLC2A6	-2.916	1.19E-06	GLUL	1.315	7.20E-06	NINJ1	0.604	5.98E-03
ASAH1	1.885	1.54E-06	CYBRD1	1.789	8.51E-06	LFNG	-0.890	6.37E-03
CXCL12	4.330	1.19E-06	C15orf48	-1.970	8.51E-06	NA	1.375	6.37E-03
NCF1	-3.423	1.76E-06	NCF1B	-3.281	7.20E-06	STX1A	-1.811	5.98E-03
GLUL	1.511	1.93E-06	CXCL8	-3.115	8.76E-06	HLA-DMB	1.485	6.37E-03
NIBAN2	-1.589	1.93E-06	EPAS1	1.630	8.76E-06	OCSTAMP	-2.205	6.37E-03
NCF1C	-3.075	1.93E-06	DPEP2	3.501	7.20E-06	GLIS3	-1.394	6.80E-03
ADA2	1.628	2.10E-06	EPHX1	2.083	1.11E-05	MS4A4A	1.146	6.80E-03
CREG1	1.862	2.31E-06	ADA2	1.320	1.15E-05	LPAR6	1.145	6.80E-03

Supplementary Table 2, related to figure 4: Top 20 differentially expressed genes between plating densities

Top 20 most significantly differentially expressed genes between plating densities. Log_2FC = Log_2(Fold change). adj.P.Val = adjusted P value. Genes consistently observed in the top 20 across all 3 density comparisons are marked in red. Those seen in the top 20 of 2 out of 3 comparisons are marked in blue.

Accepted Description	Conc.in media (M)	Adducts	m/z	Retention time (min)	Mean Abundance				Standard Deviation			
					Media	Low	Mid	High	Media	Low	Mid	High
Alanine	5.0E-05	M+H	260.103	5.064	1.36E+04	1.09E+05	1.10E+05	1.15E+05	3.30E+02	1.80E+03	3.17E+03	1.73E+03
Arginine	7.0E-04	M+H	345.167	3.394	4.74E+04	4.41E+04	4.04E+04	4.21E+04	1.31E+03	1.38E+03	1.01E+03	1.04E+03
Asparagine	5.0E-05	M+H	303.109	2.583	6.98E+03	5.68E+03	4.65E+03	5.87E+03	3.24E+02	1.28E+02	5.32E+01	1.33E+02
Aspartic acid*	5.0E-05	N/A	N/A	N/A	3.44E+09	2.45E+08	2.48E+08	2.32E+08	2.79E+08	2.43E+07	4.12E+07	2.43E+07
Glutamic acid	5E-05	M+H	318.108	4.296	1.97E+03	2.15E+02	2.76E+02	2.86E+02	1.69E+02	3.01E+01	1.05E+03	1.09E+01
Glutamic acid*	5.0E-05	N/A	N/A	N/A	4.02E+09	3.23E+09	3.24E+09	3.58E+09	3.11E+08	3.39E+08	2.29E+08	1.94E+08
Glutamine**	2.0E-03	M+H	317.125	3.343	1.69E+02	6.29E+04	6.74E+04	7.33E+04	1.41E+01	1.87E+03	1.99E+03	1.81E+03
Glycine	2.5E-04	M+H	246.087	3.502	2.14E+04	2.90E+04	2.99E+04	3.24E+04	4.51E+02	1.14E+03	5.53E+02	7.07E+02
Histidine	1.5E-04	M+H	326.125	2.273	1.15E+04	1.17E+04	1.12E+04	1.22E+04	2.39E+02	3.56E+02	1.29E+02	1.90E+02
Isoleucine	4.2E-04	M+H	302.150	7.890	8.62E+04	8.13E+04	7.31E+04	7.90E+04	2.09E+03	2.55E+03	1.83E+03	1.62E+03
Leucine	4.5E-04	M+H	302.150	7.806	7.56E+04	6.75E+04	6.36E+04	6.66E+04	2.02E+03	1.98E+03	1.69E+03	1.95E+03
Lysine	5.0E-04	M+H	487.210	6.661	8.96E+04	8.99E+04	8.82E+04	9.30E+04	1.15E+03	1.66E+03	1.68E+03	1.41E+03
Phenylalanine	2.2E-04	M+H	336.135	7.998	5.86E+04	6.11E+04	5.82E+04	6.36E+04	1.12E+03	1.99E+03	9.62E+02	1.11E+03
Proline	1.5E-04	M+H	286.119	5.666	2.90E+04	3.06E+04	2.98E+04	3.41E+04	9.38E+02	1.40E+03	1.36E+03	2.95E+02
Serine	2.5E-04	M+H	276.098	3.226	1.96E+04	8.24E+03	2.32E+03	2.69E+03	4.84E+02	1.84E+02	5.77E+02	2.03E+02
Threonine	4.5E-04	M+H	290.114	4.689	4.48E+04	4.73E+04	4.58E+04	4.83E+04	1.30E+03	1.23E+03	1.25E+03	1.24E+03
Tryptophan	4.4E-05	M+H	375.145	8.099	1.29E+04	1.22E+04	1.12E+04	1.29E+04	2.66E+02	4.31E+02	4.20E+02	4.15E+02
Tyrosine	0.000214	M+H	352.1296	6.778	3.73E+04	3.77E+04	3.68E+04	3.85E+04	3.64E+02	9.56E+02	4.57E+02	9.97E+02
Valine	0.000451	M+H	288.1344	7.062	6.76E+04	6.66E+04	6.32E+04	6.62E+04	7.73E+02	1.44E+03	1.68E+03	6.32E+02

Supplementary table 3, related to figure 5: Amino acid abundance in the supernatant

Abundance of derivatised amino acids (with the exception of Cysteine and Methionine) in the supernatant of low, mid, and high density cells compared to a fresh media control, as determined by LC-MS. * Abundance of aspartic acid and glutamic acid was also determined from non-derivatised samples. ** Glutamine is supplemented into the media in a precursor dipeptide L-alanyl-L-glutamine form which is converted by cells into L-glutamine hence low abundance in the media control. Mean abundance shown calculated from 6 experimental replicates.

Name	Formula	Annot. ΔMass [ppm]	Calc. MW	RT [min]	Area under the curve			
					Media	Low	Mid	High
(2R)-2,3-Dihydroxypropanoic acid	C3 H6 O4	0.23	106.0266	4.113	1.92E+08	5.50E+08	5.33E+08	1.26E+09
2-Deoxyribose 5-phosphate	C5 H11 O7 P	0.67	214.0244	10.124	3.58E+05	2.74E+07	2.80E+07	1.83E+07
2-Hydroxy-2-methylbutyric acid	C5 H10 O3	0.8	118.0631	5.441	2.57E+06	1.15E+08	2.51E+08	1.05E+09
2-Hydroxybutyric acid	C4 H8 O3	0.58	104.0474	4.299	7.97E+06	8.08E+08	1.25E+09	1.03E+09
2-Methylbenzoic acid	C8 H8 O2	0.77	136.0525	14.756	5.16E+05	4.09E+07	7.84E+07	1.37E+08
2-Oxoglutaric acid	C5 H6 O5	0.38	146.0216	11.518	3.69E+06	1.62E+08	2.78E+08	4.05E+08
3-Methyl-2-oxovaleric acid	C6 H10 O3	0.72	130.0631	9.897	7.26E+06	5.55E+09	4.13E+09	3.08E+09
3-Methyl-2-oxovaleric acid	C6 H10 O3	0.74	130.0631	9.42	6.94E+06	6.10E+09	4.44E+09	3.41E+09
4-Hydroxy-3-methoxymandelic acid	C9 H10 O5	0.47	198.0529	14.76	5.33E+05	1.13E+07	2.27E+07	3.69E+07
4-Hydroxyproline	C5 H9 N O3	0.48	131.0583	9.174	8.63E+06	7.71E+07	1.16E+08	1.32E+08
4-Oxoproline	C5 H7 N O3	0.62	129.0427	3.909	8.45E+07	1.24E+11	1.08E+11	5.54E+10
5-Aminolevulinic acid	C5 H9 N O3	-0.02	131.0582	3.283	1.14E+06	9.16E+06	2.13E+07	3.42E+07
6-Hydroxycaproic acid	C6 H12 O3	0.6	132.0787	6.825	6.26E+06	8.06E+07	1.39E+08	4.10E+08
Acetoacetate	C4 H6 O3	-0.13	102.0317	6.884	1.41E+07	3.91E+07	2.59E+07	9.45E+06
Acetylcysteine	C5 H9 N O3 S	0.01	163.0303	12.271	8.33E+07	2.93E+07	2.69E+07	3.36E+07
Aconitic acid	C6 H6 O6	-0.54	174.0163	15.287	1.80E+07	1.66E+08	2.65E+08	3.93E+08
Arabinose	C5 H10 O5	-0.14	150.0528	3.729	5.32E+07	4.93E+07	3.85E+07	2.08E+07
Ascorbate	C6 H8 O6	-0.48	176.032	10.999	4.23E+08	3.30E+08	3.11E+08	3.32E+08
Aspartate	C4 H7 N O4	0.29	133.0376	10.208	1.18E+09	9.63E+06	1.17E+07	9.74E+06
cis-Aconitic acid	C6 H6 O6	-0.65	174.0163	16.378	1.38E+07	9.00E+07	1.88E+08	4.55E+08
Citric acid	C6 H8 O7	0.08	192.027	15.287	2.29E+09	1.98E+10	3.16E+10	4.72E+10
D-(+)-Galactose	C6 H12 O6	0.04	180.0634	3.695	9.65E+08	6.14E+08	8.68E+08	1.26E+09
D-Glucose 6-phosphate	C6 H13 O9 P	0.35	260.0298	11.147	2.65E+06	2.89E+07	6.10E+07	1.31E+08
dl-3-Indolelactic acid	C11 H11 N O3	0.31	205.074	23.762	9.55E+05	3.71E+07	6.93E+07	1.96E+08
DL-Malic acid	C4 H6 O5	0.64	134.0216	10.07	7.84E+06	5.04E+08	7.54E+08	1.17E+09
D-Ribulose 5-phosphate	C5 H11 O8 P	0.37	230.0192	11.918	2.57E+06	1.49E+07	2.76E+07	3.73E+07
D-Sedoheptulose 7-phosphate	C7 H15 O10 P	1.11	290.0406	12.076	3.92E+05	1.53E+07	3.78E+07	9.46E+07
D-Xylulosonic acid	C5 H8 O6	0.43	164.0322	5.783	6.26E+06	8.00E+07	5.46E+07	3.25E+07
D-α-Hydroxyglutaric acid	C5 H8 O5	0.7	148.0373	9.86	9.58E+06	6.38E+07	9.52E+07	1.73E+08
Fumaric acid	C4 H4 O4	0.35	116.011	12.121	2.64E+06	6.34E+07	9.77E+07	1.70E+08
Galactaric acid (mucic acid)	C6 H10 O8	0.46	210.0377	9.886	1.90E+07	6.68E+07	6.08E+07	6.50E+07
Galacturonic acid	C6 H10 O7	0.35	194.0427	5.271	2.15E+07	1.18E+08	8.64E+07	7.48E+07
Gluconic acid	C6 H12 O7	0.25	196.0584	3.804	8.75E+08	2.94E+09	2.37E+09	1.99E+09
Glucose 1-phosphate	C6 H13 O9 P	0.62	260.0299	8.813	9.32E+05	1.86E+07	3.25E+07	3.69E+07
Glutamylcysteine	C8 H14 N2 O5 S	1.04	250.0626	14.898	1.26E+06	1.04E+07	2.34E+07	2.68E+07
Glutathione (GSH)	C10 H17 N3 O6 S	1.59	307.0843	14.444	6.66E+07	1.20E+08	1.68E+08	1.83E+08
Glyceric acid	C3 H6 O4	-0.45	106.0266	5.821	5.92E+10	5.78E+09	5.20E+09	2.47E+09
Glycerol 3-phosphate	C3 H9 O6 P	-0.25	172.0136	8.839	2.26E+06	3.06E+07	5.83E+07	2.25E+08
Glycolic acid	C2 H4 O3	-0.42	76.01601	4.38	6.63E+07	2.38E+08	2.57E+08	7.85E+07
Gly-l-pro	C7 H12 N2 O3	0.13	172.0848	6.241	9.74E+06	3.51E+07	1.76E+07	1.30E+07

Guanosine monophosphate	C10 H14 N5 O8 P	1.42	363.0585	19.294	1.81E+06	5.28E+06	1.26E+07	2.61E+07
Î±-L-Glutamyl-L-glutamine	C10 H17 N3 O6	1.01	275.112	9.345	3.83E+06	3.82E+08	6.91E+08	1.06E+09
Isobutyric acid	C4 H8 O2	0.06	88.05244	5.345	4.46E+06	2.48E+07	3.29E+07	2.40E+07
Itaconic acid	C5 H6 O4	0.93	130.0267	16.378	7.29E+06	3.69E+07	7.67E+07	1.82E+08
L-(-)-3-Phenyllactic acid	C9 H10 O3	-0.11	166.063	11.097	8.56E+05	2.31E+08	4.59E+08	1.00E+09
L-(+)-Lactic acid	C3 H6 O3	0.18	90.03171	3.763	2.98E+08	2.46E+10	1.92E+10	6.13E+10
Lactoyl-isoleucine	C9 H17 N O4	0.2	203.1158	6.883	8.79E+05	5.36E+06	1.39E+07	7.37E+07
L-Threonic acid	C4 H8 O5	0.53	136.0372	3.554	9.68E+07	3.49E+08	4.23E+08	4.81E+08
Methyl acetoacetate	C5 H8 O3	0.52	116.0474	7.513	2.07E+06	1.29E+09	1.04E+09	7.87E+08
Methylmalonic acid	C4 H6 O4	0.74	118.0267	10.039	1.23E+07	2.41E+08	2.92E+08	3.54E+08
Mevalonic acid	C6 H12 O4	0.61	148.0737	5.046	1.73E+08	2.25E+08	2.02E+08	2.40E+08
N-Acetyl-1-aspartylglutamic acid	C11 H16 N2 O8	1.3	304.0911	13.17	4.70E+05	2.68E+07	5.03E+07	7.45E+07
N-Acetyl-aspartate	C6 H9 N O5	-0.43	175.048	9.175	3.32E+06	1.02E+09	1.56E+09	1.78E+09
N-Acetylneuraminic acid (Sialic acid)	C11 H19 N O9	1.31	309.1064	3.41	4.56E+07	1.19E+10	1.06E+10	9.76E+09
Neopterin	C9 H11 N5 O4	2.33	253.0817	3.837	7.01E+06	1.41E+08	1.17E+08	6.29E+07
N-Formyl-methionine	C6 H11 N O3 S	0.31	177.046	5.921	3.42E+06	1.14E+07	1.83E+07	2.68E+07
O-Phosphorylethanolamine	C2 H8 N O4 P	0.67	141.0192	10.697	2.98E+06	5.11E+08	7.58E+08	1.04E+09
Oxalic acid	C2 H2 O4	0.23	89.99533	11.976	1.07E+08	1.97E+08	2.03E+08	2.15E+08
Palmitic acid	C16 H32 O2	0.66	256.2404	35.644	1.51E+09	1.60E+09	1.87E+09	7.88E+08
Pantothenic acid	C9 H17 N O5	0.38	219.1108	4.189	4.14E+09	4.42E+09	3.92E+09	5.60E+09
Phenylacetaldehyde	C8 H8 O	0.31	120.0576	14.753	3.67E+05	1.42E+07	2.67E+07	4.71E+07
Phenylacetylglycine	C10 H11 N O3	0.07	193.0739	9.77	1.17E+09	8.68E+08	8.05E+08	8.12E+08
Phenylpyruvic acid	C9 H8 O3	0.36	164.0474	25.859	1.84E+06	2.08E+08	2.42E+08	1.56E+08
Pseudouridine	C9 H12 N2 O6	0.38	244.0696	6.415	1.09E+06	5.77E+07	1.05E+08	1.69E+08
Pyrogallol-2-O-sulphate	C6 H6 O6 S	0.38	205.9886	11.608	1.03E+06	2.83E+08	4.75E+08	6.65E+08
Pyruvic acid	C3 H4 O3	-0.12	88.01603	15.288	3.87E+07	3.99E+08	6.52E+08	8.95E+08
Ribulose 5-phosphate	C5 H11 O8 P	0.62	230.0193	8.987	1.78E+05	1.86E+07	2.01E+07	2.12E+07
Taurine	C2 H7 N O3 S	0.23	125.0147	4.348	2.07E+06	4.67E+07	6.40E+07	1.92E+08
Uracil	C4 H4 N2 O2	0.64	112.0274	7.56	9.74E+05	1.17E+07	8.29E+06	2.95E+07
Uridine 5'-monophosphate	C9 H13 N2 O9 P	1.46	324.0363	16.739	1.55E+06	1.46E+07	2.56E+07	3.86E+07

Supplementary table 4, related to figure 5: Extracellular metabolites putatively identified by LC-MS

Complete list of metabolites in the supernatant are measured by LC-MS and putatively identified using Compound Discoverer software based on online and in-house standard libraries. RT = retention time. Area under the curve shown are mean values calculated from 6 experimental replicates.

Gene	Forward Primer	Reverse Primer	Reference
ADA2	ATGGGGCTCCGAATCAAGTTC	CTTGTAGTCATGCAAGGAGTGG	(44)
C1QA	GTGACACATGCTCTAAGAAG	GACTCTTAAGCACTGGATTG	(45)
CCL3	AGTTCTCTGCATCACTTGCTG	CGGCTTCGCTTGGTTAGGAA	(44)
CCL5	CCAGCAGTCGTCTTTGTCAC	CTCTGGGTTGGCACACACTT	(44)
CR1	AGAGGGACGAGCTTCGACC	TCAGGACGGCATTCGTACTTT	(44)
CX3CR1	TGACTGGCAGATCCAGAGGTT	TTCTGTCACTGATTCAGGGAACTG	(46)
CXCL11	GACGCTGTCTTTGCATAGGC	GGATTTAGGCATCGTTGTCCTTT	(44)
CXCL12	ATTCTCAACACTCCAAACTGTGC	ACTTTAGCTTCGGGTCAATGC	(44)
FASN	GTCTCTGAAGGGCATCCTGG	TCGTGTTGACTTCTCGCTCC	(46)
FCGR2B	AGCCAATCCCACTAATCCTGA	GGTGCATGAGAAGTGAATAGGTG	(44)
GAPDH	TGGACCTGACCTGCCGTCTA	CCCTGTTGCTGTAGCCAAATTC	(47)
GLUL	TAAGGACCCTAACAAGCTGGT	CCGTTTACAGGTGTGCCTCAA	(44)
HK1	GCAGCTCCTGGCCTATTACT	TGGCATAGAGATACTTGTCAATCT	(46)
HLA-DMB	TTCTGGGGATCACTGACCAAC	GGCTCCCTCGTGTTAAAAGGA	(44)
IL10	TCAAGGCGCATGTGAACTCC	GATGTCAAACTCACTCATGGCT	(44)
IL1B	AGCTACGAATCTCCGACCAC	CGTTATCCCATGTGTCGAAGAA	(44)
LC3A	CCAGCAAAATCCCGGTGAT	CCGGATGATCTTGACCAACTC	(44)
MMP9	TGTACCGCTATGGTTACACTCG	GGCAGGGACAGTTGCTTCT	(44)
TGFB	CAATTCCTGGCGATACCTCAG	GCACAACTCCGGTGACATCAA	(44)
TNFA	GGACCTCTCTCTAATCAGCCCTC	TCGAGAAGATGATCTGACTGCC	(44)
UBE4A	TAGCCGCTCATTCCGATCAC	GGGATGCCATTCCCGCTTT	(46)
VIPAR	GGGAGACCCAAAGGGGAGTAT	GGAGCGGAATCTCTCTAGTGAG	(46)

Supplementary table 5, related to Methods: Primers used for qPCR

Stage	Temp. (ºC)	Time (min:sec)	Ramp rate (ºC/s)	Cycles
Hold	50	02:00	1.6	1
	95	10:00	1.6	
PCR	95	00:15	1.6	40
	60	01:00	1.6	
Melt Curve	95	00:15	1.6	1
	60	01:00	1.6	
	95	00:01	0.075	

Supplementary Table 6, related to methods: qPCR Cycling conditions

3. Supplementary References:

1. Vaughan-Jackson A, Stodolak S, Ebrahimi KH, Browne C, Reardon PK, Pires E, et al. Differentiation of human induced pluripotent stem cells to authentic macrophages using a defined, serum-free, open-source medium. Stem Cell Reports. 2021;16(7):1735–48.

2. Kondo T, Kawai T, Akira S. Dissecting negative regulation of Toll-like receptor signaling. Trends Immunol [Internet]. 2012;33(9):449–58. Available from: http://dx.doi.org/10.1016/j.it.2012.05.002

3. Postat J, Olekhnovitch R, Lemaître F, Bousso P. A Metabolism-Based Quorum Sensing Mechanism Contributes to Termination of Inflammatory Responses. Immunity. 2018;49(4):654-665.e5.

4. Reich M, Paris I, Ebeling M, Dahm N, Schweitzer C, Reinhardt D, et al. Alzheimer's Risk Gene TREM2 Determines Functional Properties of New Type of Human iPSC-Derived Microglia. Front Immunol. 2021;11(February):1–15.

5. Boulakirba S, Pfeifer A, Mhaidly R, Obba S, Goulard M, Schmitt T, et al. IL-34 and CSF-1 display an equivalent macrophage differentiation ability but a different polarization potential. Sci Rep. 2018;8(1):1–11.

6. Graziano F, Aimola G, Forlani G, Turrini F, Accolla RS, Vicenzi E, et al. Reversible Human Immunodeficiency Virus Type-1 Latency in Primary Human Monocyte-Derived Macrophages Induced by Sustained M1 Polarization. Sci Rep [Internet]. 2018;8(1):1–13. Available from: http://dx.doi.org/10.1038/s41598-018-32451-w

7. Smith SR, Schaaf K, Rajabalee N, Wagner F, Duverger A, Kutsch O, et al. The phosphatase PPM1A controls monocyte-to-macrophage differentiation. Sci Rep. 2018;8(1):1–14.

8. Zhang S, Shrestha CL, Kopp BT. Cystic fibrosis transmembrane conductance regulator (CFTR) modulators have differential effects on cystic fibrosis macrophage function. Sci Rep [Internet]. 2018;8(1):1–10. Available from: http://dx.doi.org/10.1038/s41598-018-35151-7

9. Eligini S, Cosentino N, Fiorelli S, Fabbiocchi F, Niccoli G, Refaat H, et al. Biological profile of monocyte-derived macrophages in coronary heart disease patients: implications for plaque morphology. Sci Rep. 2019;9(1):1–14.

10. Geiß C, Alanis-Lobato G, Andrade-Navarro M, Régnier-Vigouroux A. Assessing the reliability of gene expression measurements in very-low-numbers of human monocyte-derived macrophages. Sci Rep. 2019;9(1):1–13.

11. Schulz D, Severin Y, Zanotelli VRT, Bodenmiller B. In-Depth Characterization of Monocyte-Derived Macrophages using a Mass Cytometry-Based Phagocytosis Assay. Sci Rep [Internet]. 2019;9(1):1–12. Available from: http://dx.doi.org/10.1038/s41598-018-38127-9

12. Wang C, Nanni L, Novakovic B, Megchelenbrink W, Kuznetsova T, Stunnenberg HG, et al. Extensive epigenomic integration of the glucocorticoid response in primary human monocytes and in vitro derived macrophages. Sci Rep. 2019;9(1):1–17.

13. da Silva CO, Gicquel T, Daniel Y, Bártholo T, Vène E, Loyer P, et al. Alteration of immunophenotype of human macrophages and monocytes after exposure to cigarette smoke. Sci Rep [Internet]. 2020;10(1):1–13. Available from: https://doi.org/10.1038/s41598-020-68753-1

14. Faria CP, Neves BM, Lourenço Á, Cruz MT, Martins JD, Silva A, et al. Giardia lamblia Decreases NF-κB p65RelA Protein Levels and Modulates LPS-Induced Pro-Inflammatory Response in Macrophages. Sci Rep. 2020;10(1):1–17.

15. Hazlett HF, Hampton TH, Aridgides DS, Armstrong DA, Dessaint JA, Mellinger DL, et al. Altered iron metabolism in cystic fibrosis macrophages: the impact of CFTR modulators and implications for Pseudomonas aeruginosa survival. Sci Rep [Internet]. 2020;10(1):1–14. Available from: https://doi.org/10.1038/s41598-020-67729-5

16. Moradian H, Roch T, Lendlein A, Gossen M. mRNA Transfection-Induced Activation of Primary Human Monocytes and Macrophages: Dependence on Carrier System and Nucleotide Modification. Sci Rep. 2020;10(1):1–15.

17. O'Regan GC, Farag SH, Ostroff GR, Tabrizi SJ, Andre R. Wild-type huntingtin regulates human macrophage function. Sci Rep [Internet]. 2020;10(1):1–12. Available from: https://doi.org/10.1038/s41598-020-74042-8

18. Ruscitti P, Di Benedetto P, Berardicurti O, Panzera N, Grazia N, Lizzi AR, et al. Pro-inflammatory properties of H-ferritin on human macrophages, ex vivo and in vitro observations. Sci Rep [Internet]. 2020;10(1):1–11. Available from: https://doi.org/10.1038/s41598-020-69031-w

19. Vrieling F, Kostidis S, Spaink HP, Haks MC, Mayboroda OA, Ottenhoff THM, et al. Analyzing the impact of Mycobacterium tuberculosis infection on primary human macrophages by combined exploratory and targeted metabolomics. Sci Rep. 2020;10(1):1–13.

20. Akhtari M, Zargar SJ, Vojdanian M, Jamshidi A, Mahmoudi M. Monocyte-derived and M1 macrophages from ankylosing spondylitis patients released higher TNF-α and expressed more IL1B in response to BzATP than macrophages from healthy subjects. Sci Rep [Internet]. 2021;11(1):1–10. Available from: https://doi.org/10.1038/s41598-021-96262-2

21. Griffoni C, Neidhart B, Yang K, Groeber-Becker F, Maniura-Weber K, Dandekar T, et al. In vitro skin culture media influence the viability and inflammatory response of primary macrophages. Sci Rep [Internet]. 2021;11(1):1–11. Available from: https://doi.org/10.1038/s41598-021-86486-7

22. Krause M, Crauwels P, Blanco-Pérez F, Globisch M, Wangorsch A, Henle T, et al. Human monocyte-derived type 1 and 2 macrophages recognize Ara h 1, a major peanut allergen, by different mechanisms. Sci Rep [Internet]. 2021;11(1):1–13. Available from: https://doi.org/10.1038/s41598-021-89402-1

23. Laverdure S, Wang Z, Yang J, Yamamoto T, Thomas T, Sato T, et al. Interleukin-27 promotes autophagy in human serum-induced primary macrophages via an mTOR- and LC3-independent pathway. Sci Rep [Internet]. 2021;11(1):1–13. Available from: https://doi.org/10.1038/s41598-021-94061-3

24. Yusuf B, Mukovozov I, Patel S, Huang YW, Liu GY, Reddy EC, et al. The neurorepellent, Slit2, prevents macrophage lipid loading by inhibiting CD36-dependent binding and internalization of oxidized low-density lipoprotein. Sci Rep [Internet]. 2021;11(1):1–13. Available from: https://doi.org/10.1038/s41598-021-83046-x

25. Choi K, Vodyanik MA, Slukvin II. Generation of mature human myelomonocytic cells through expansion and differentiation of pluripotent stem cell–derived lin–CD34+CD43+CD45+ progenitors. J Clin Invest. 2009;119(9):2818.

26. Senju S, Haruta M, Matsunaga Y, Fukushima S, Ikeda T, Takahashi K, et al. Characterization of dendritic cells and macrophages generated by directed differentiation from mouse induced pluripotent stem cells. Stem Cells. 2009;27(5):1021–31.

27. Choi KD, Vodyanik M, Slukvin II. Hematopoietic differentiation and production of mature myeloid cells from human pluripotent stem cells. Nat Protoc. 2011;6(3):296–313.

28. Kambal A, Mitchell G, Cary W, Gruenloh W, Jung Y, Kalomoiris S, et al. Generation of HIV-1 resistant and functional macrophages from hematopoietic stem cell-derived induced pluripotent stem cells. Mol Ther [Internet]. 2011;19(3):584–93. Available from: http://dx.doi.org/10.1038/mt.2010.269

29. Salvagiotto G, Burton S, Daigh CA, Rajesh D, Slukvin II, Seay NJ. A defined, feeder-free, serum-free system to generate In Vitro hematopoietic progenitors and differentiated blood cells from hESCs and hiPSCs. PLoS One. 2011;6(3).

30. Senju S, Haruta M, Matsumura K, Matsunaga Y, Fukushima S, Ikeda T, et al. Generation of dendritic cells and macrophages from human induced pluripotent stem cells aiming at cell therapy. Gene Ther. 2011;18(9):874–83.

31. van Wilgenburg B, Browne C, Vowles J, Cowley SA. Efficient, Long Term Production of Monocyte-Derived Macrophages from Human Pluripotent Stem Cells under Partly-Defined and Fully-Defined Conditions. PLoS One. 2013;8(8).

32. Yanagimachi MD, Niwa A, Tanaka T, Honda-Ozaki F, Nishimoto S, Murata Y, et al. Robust and Highly-Efficient Differentiation of Functional Monocytic Cells from Human Pluripotent Stem Cells under Serum- and Feeder Cell-Free Conditions. PLoS One. 2013;8(4):1–9.

33. Brault J, Goutagny E, Telugu N, Shao K, Baquié M, Satre V, et al. Optimized generation of functional neutrophils and macrophages from patient-specific induced pluripotent stem cells: Ex vivo models of X0-Linked, AR220- and AR470-chronic granulomatous diseases. Biores Open Access. 2014;3(6):311–26.

34. Lachmann N, Ackermann M, Frenzel E, Liebhaber S, Brennig S, Happle C, et al. Large-scale hematopoietic differentiation of human induced pluripotent stem cells provides granulocytes or macrophages for cell replacement therapies. Stem Cell Reports. 2015;4(2):282–96.

35. Schwartz MP, Hou Z, Propson NE, Zhang J, Engstrom CJ, Costa VS, et al. Human pluripotent stem cell-derived neural constructs for predicting neural toxicity. Proc Natl Acad Sci U S A. 2015;112(40):12516–21.

36. Takata K, Kozaki T, Lee CZW, Thion MS, Otsuka M, Lim S, et al. Induced-Pluripotent-Stem-Cell-Derived Primitive Macrophages Provide a Platform for Modeling Tissue-Resident Macrophage Differentiation and Function. Immunity. 2017;47(1):183-198.e6.

37. Ackermann M, Kempf H, Hetzel M, Hesse C, Hashtchin AR, Brinkert K, et al. Bioreactor-based mass production of human iPSC-derived macrophages enables immunotherapies against bacterial airway infections. Nat Commun. 2018;9(1):1–13.

38. Hansen M, Varga E, Aarts C, Wust T, Kuijpers T, von Lindern M, et al. Efficient production of erythroid, megakaryocytic and myeloid cells, using single cell-derived iPSC colony differentiation. Stem Cell Res [Internet]. 2018;29(April):232–44. Available from: https://doi.org/10.1016/j.scr.2018.04.016

39. Mukherjee C, Hale C, Mukhopadhyay S. A Simple Multistep Protocol for Differentiating Human Induced Pluripotent Stem Cells into Functional Macrophages. In: Rousselet G, editor.

Macrophages: Methods and Protocols [Internet]. New York, NY: Springer New York; 2018. p. 13–28. Available from: https://doi.org/10.1007/978-1-4939-7837-3_2

40. Zhang H, Xue C, Shah R, Bermingham K, Hinkle CC, Li W, et al. Functional Analysis and Transcriptomic Profiling of iPSC-Derived Macrophages and Their Application in Modeling Mendelian Disease. Circ Res. 2015;117(1):17–28.

41. Cao X, Yakala GK, van den Hil FE, Cochrane A, Mummery CL, Orlova V V. Differentiation and Functional Comparison of Monocytes and Macrophages from hiPSCs with Peripheral Blood Derivatives. Stem Cell Reports [Internet]. 2019;12(6):1282–97. Available from: https://doi.org/10.1016/j.stemcr.2019.05.003

42. Gutbier S, Wanke F, Dahm N, Rümmelin A, Zimmermann S, Christensen K, et al. Large-scale production of human IPSC-derived macrophages for drug screening. Int J Mol Sci. 2020 Jul 1;21(13):1–23.

43. Monkley S, Krishnaswamy JK, Goransson M, Clausen M, Mueller J, Thorn K, et al. Optimised generation of iPSC-derived macrophages and dendritic cells that are functionally and transcriptionally similar to their primary counterparts. PLoS One. 2020;1–17.

44. Wang X, Spandidos A, Wang H, Seed B. PrimerBank: A PCR primer database for quantitative gene expression analysis, 2012 update. Nucleic Acids Res. 2012;40(D1):1144–9.

45. Haenseler W, Sansom SN, Buchrieser J, Newey SE, Moore CS, Nicholls FJ, et al. A Highly Efficient Human Pluripotent Stem Cell Microglia Model Displays a Neuronal-Co-culture-Specific Expression Profile and Inflammatory Response. Stem Cell Reports [Internet]. 2017;8(6):1727–42. Available from: http://dx.doi.org/10.1016/j.stemcr.2017.05.017

46. Ye J, Coulouris G, Zaretskaya I, Cutcutache I, Rozen S, Madden TL. Primer-BLAST: a tool to design target-specific primers for polymerase chain reaction. BMC Bioinformatics. 2012;13(134):134.

47. Zhang X, Ding L, Sandford AJ. Selection of reference genes for gene expression studies in human neutrophils by real-time PCR. BMC Mol Biol. 2005;6:1–7.
